# Dynamic Expression and Regulatory Network of Circular RNA for Abdominal Preadipocytes Differentiation in Chicken (*Gallus gallus*)

**DOI:** 10.3389/fcell.2021.761638

**Published:** 2021-11-12

**Authors:** Weihua Tian, Bo Zhang, Haian Zhong, Ruixue Nie, Yao Ling, Hao Zhang, Changxin Wu

**Affiliations:** ^1^ National Engineering Laboratory for Animal Breeding, College of Animal Science and Technology, China Agricultural University, Beijing, China; ^2^ Beijing Key Laboratory for Animal Genetic Improvement, College of Animal Science and Technology, China Agricultural University, Beijing, China

**Keywords:** chicken, circRNA, abdominal fat, adipogenic differentiation, competing endogenous RNA

## Abstract

Circular RNA (circRNA), as a novel endogenous biomolecule, has been emergingly demonstrated to play crucial roles in mammalian lipid metabolism and obesity. However, little is known about their genome-wide identification, expression profile, and function in chicken adipogenesis. In present study, the adipogenic differentiation of chicken abdominal preadipocyte was successfully induced, and the regulatory functional circRNAs in chicken adipogenesis were identified from abdominal adipocytes at different differentiation stages using Ribo-Zero RNA-seq. A total of 1,068 circRNA candidates were identified and mostly derived from exons. Of these, 111 differentially expressed circRNAs (DE-circRNAs) were detected, characterized by stage-specific expression, and enriched in several lipid-related pathways, such as Hippo signaling pathway, mTOR signaling pathway. Through weighted gene co-expression network analyses (WGCNA) and K-means clustering analyses, two DE-circRNAs, Z:35565770|35568133 and Z:54674624|54755962, were identified as candidate regulatory circRNAs in chicken adipogenic differentiation. Z:35565770|35568133 might compete splicing with its parental gene, *ABHD17B*, owing to its strictly negative co-expression. We also constructed competing endogenous RNA (ceRNA) network based on DE-circRNA, DE-miRNA, DE-mRNAs, revealing that Z:54674624|54755962 might function as a ceRNA to regulate chicken adipogenic differentiation through the gga-miR-1635-*AHR2*/*IRF1*/*MGAT3*/*ABCA1*/*AADAC* and/or the novel_miR_232-*STAT5A* axis. Translation activity analysis showed that Z:35565770|35568133 and Z:54674624|54755962 have no protein-coding potential. These findings provide valuable evidence for a better understanding of the specific functions and molecular mechanisms of circRNAs underlying avian adipogenesis.

## Introduction

Excessive body fat accumulation has been triggered by the overemphasis on genetic selection for growth rate and feed conversion in broilers and laying hens, especially abdominal fat accumulation, consequently leading to significantly reduced feed utilization, eggs performance, fertilization rate and hatchability, increased difficulty of meat product processing, coupled with enhanced nitrogen and phosphate content in excrement, causing serious pollution to the ecological environment (Geraert et al., 1990; Zhang X. Y. et al., 2018). It has notably become one of the main problems to be solved for the broiler and layer breeding industry. Therefore, it is of great significance to in-depth study the mechanism of body fat deposition and molecular regulatory mechanisms.

It is generally acknowledged that abdominal fat deposition grew by both hyperplasia (number) and hypertrophy (size) of abdominal adipocyte, wherein hyperplasia depended upon the proliferation of preadipocytes, which has been fixed in the embryonic stage and early growth and development; the deposition of abdominal fat in the late growth and development principally relies on the differentiation of preadipocytes into mature adipocytes. Therefore, abdominal fat deposition refers to a complex and precisely orchestrated process involving a network of genes, transcriptional factors, even epigenetic modification and regulation, for instance, non-coding RNAs (ncRNA) containing circular RNA (circRNA), long-chain non-coding RNA (lncRNA) and microRNA (miRNA), which has been gradually realized to exert crucial roles in various biological processes through regulating gene expression. The circRNAs were originally considered as “junk” generated by aberrant splicing events. However, emerging evidences have supported that circRNA could serve as a novel and endogenous RNA modulator that functions in many diseases, growth and development, lipid metabolism in mammals as well as a new and highly effective molecular marker in the diagnosis and treatment of cancer, obesity, cardiovascular diseases, etc (Han et al., 2018). A recent study pointed to the potential that circRNAs turned into a type of new and high effective vaccine and treatments strategy, with the advent of circRNA vaccines against SARS-CoV-2 (Qu et al., 2021). As a class of endogenous RNA, circRNA is derived from liner RNA, which formed a covalently closed loop via backsplicing, but more stable than liner RNA, as a result of lacking a 5′-cap structure or 3′-polyadenylated tail, which resist RNA exonucleases or RNase R (Jeck and Sharpless, 2014). Interestingly, circRNAs harbour tissue-, cell- and developmental stage-specific expression profile, thus indicating their potential regulatory roles (Memczak et al., 2013; Salzman et al., 2013). CircRNAs are widely perceived to cis or trans regulate gene expression level via diverse mechanisms, including sponging miRNA, scaffolding protein, modulating their parental gene transcription by interacting with transcription complexes, competing with precursor mRNA (pre-mRNA) splicing, and encoding protein or peptide, eventually conducing its role in various biological processes (Han et al., 2018; Kristensen et al., 2019).

In mammals, a few circRNAs have been functionally and mechanistically characterized towards lipid metabolism. Adipose-expressed circRNAs from human visceral and subcutaneous fat tissue, as well as mouse epididymal and inguinal fat have been discovered by deep sequencing, most of which were conserved, tissue specific and dynamically regulated during adipogenesis and obesity. Dramatically, circTshz2-1 and circArhgap5-2 could induce the adipocyte differentiation and promote lipid droplet accumulation as indispensable regulators during adipogenesis and obesity (Arcinas et al., 2019). A recent study showing the comprehensive expression profile of circRNAs in the liver of obese mouse’ offspring revealed that hepatic circRNA functioned as competing endogenous RNA (ceRNA) to sponge miRNA, in turn to alleviate their silencing on target concerning lipid metabolism, ultimately responding to metabolic disorders and obesity (Chen et al., 2020). circRNA_0046366 and circRNA_0046367 was validated to prevent the hepatotoxicity of steatosis-related lipid peroxidation through circRNA_0046366 and circRNA_0046367/miR-34a/PPARα axis, which stimulated transcriptional activation of genes associated with lipid metabolism (Guo et al., 2017; Guo et al., 2018). CircRNA_002581 knockdown significantly attenuated lipid droplet accumulation and could sponge miR-122 to derepress its inhibition of target gene, cytoplasmic polyadenylation element-binding protein 1 (*CPEB1*), which might subsequently affect autophagy, thereby aggravating the progression of nonalcoholic steatohepatitis (NASH) (Jin et al., 2020).

With regard to livestock and poultry, some circRNAs have also been identified by high throughput sequencing and confirmed to participate in regulating their important biological process, such as embryos development (Veno et al., 2015), formation of skeletal muscle fiber (Li et al., 2020b), hepatic lipid metabolism (Huang et al., 2018), and adipogenic differentiation (Li A. et al., 2018) in pig; muscle growth and development (Wei et al., 2017; Li et al., 2018b; Li et al., 2018c; Liu et al., 2020; Shen et al., 2020; Yue et al., 2020; Elnour et al., 2021), testis development (Gao et al., 2018), adipogenesis (Jiang et al., 2020) and milk fat content (Chen et al., 2021) in cattle; fat deposition in buffalo and yak (Huang et al., 2019; Wang H. et al., 2020); intramuscular fat (IMF) content denoting a crucial indicator of meat quality in donkey (Li et al., 2020a); follicular development (Shen et al., 2019), Marek’s tumourigenesis (Wang L. et al., 2020) as well as muscle growth development (Ouyang et al., 2018a; Ouyang et al., 2018b) in chicken. And it has yet indicated some functional circRNAs related to lipid metabolism, for instance, circFUT10 could promote bovine preadipocyte proliferation but inhibit adipocyte differentiation (Jiang et al., 2020); circRNA_26852 and circRNA_11897, might get involved in porcine adipocyte differentiation and lipid metabolism (Li A. et al., 2018); circ11103 could increase the triglyceride levels in bovine mammary epithelial cells and the contents of unsaturated fatty acids (Chen et al., 2021); additionally, 19:45387150|45389986 and 21:6969877|69753491, were deemed as potential modulators of buffalo back subcutaneous adipose deposition (Huang et al., 2019). These evidences intensively reveal an indispensable role of circRNA in lipid metabolism in livestock. However, in chicken, very limited investigations were available about the identification, expression profiles and functions of circRNAs in lipid metabolism, especially adipogenesis in chicken, which remains to be systematically explored.

Here, we comprehensively investigated the characterization and expression profiles of circRNA, miRNA, mRNA in abdominal adipocyte during different differentiation stages, including 0, 12, 48, 72, 120 h using Ribo-Zero RNA-seq, and explored the potential functions and regulatory mechanisms of circRNA underlying chicken abdominal adipocyte differentiation. The results could provide an insight to understanding the regulatory mechanisms of adipocyte differentiation and abdominal fat deposition in chickens.

## Materials and Methods

### Ethics Statement

All experimental procedures were conducted according to the guidelines of the animal welfare committee of the State Key Laboratory for Agro-Biotechnology of the China Agricultural University (approval number, XK257).

### Chicken Preadipocyte Culture and Adipogenic Differentiation Assay

Immortalized chicken preadipocytes 2 (ICP2) from abdominal adipose tissue of 10-day-old AA broiler chickens (Wang et al., 2017) were obtained from the Key Laboratory of Chicken Genetics and Breeding, Ministry of Agriculture (Northeast Agricultural University). ICP2 cells were maintained in a basal medium consisting of Dulbecco’s modified Eagle’s medium F12 (DMEM-F12) (Gibco, Gaithersburg, MD, United States) supplemented with 10% fetal bovine serum (Gibco) and 2% penicillin-streptomycin (Gibco). While growing to 80–90% confluence, ICP2 cells were cultured in 6-well plates at an adjusted density of 1 × 10^5^ cells/mL at 37°C with 5% CO_2_ in a humidified incubator. Then, the ICP2 cells were divided into eight groups (*n* = 10 per group) and treated with a differentiation medium composed of basal DMEM-F12 medium supplemented with a final concentration of 160 μM sodium oleate (Sigma, St. Louis, MO, United States) dissolved in sterile deionized water to induce adipogenesis. The differentiation medium was changed every day, and the cells were washed in phosphate-buffered saline (PBS) three times and harvested after 0, 6, 12, 24, 48, 72, 96, and 120 h (*n* = 6 per group). The samples were stored at −80°C until further use. In addition, adipocyte differentiation was examined via Oil Red O staining (*n* = 3) and boron-dipyrromethene (BODIPY) (Invitrogen, Carlsbad, CA, United States) fluorescent dye staining.

### Oil Red O Staining

The cells were washed with PBS three times and fixed in 10% formaldehyde for 30 min at 25°C. The cells were washed three times with PBS and stained with Oil Red O (Sigma) for 30 min. After incubation with 60% isopropyl alcohol for 10 s, the cells were washed with PBS three times and imaged under a microscope (Leica, Bensheim, Germany). Subsequently, 100% isopropyl alcohol was used to dissolve intracellular Oil Red O staining to assess the content of lipid droplets using spectrophotometry at 490 nm.

### BODIPY Fluorescent Staining

The cells were washed with PBS three times and fixed in 10% formaldehyde for 30 min at room temperature. Next, the cells were washed with PBS and incubated with fluorescent dye BODIPY®493/503 (4,4-difluoro-3a,4a-diaza-s-indacene) (Invitrogen) diluted in PBS (1:1000) dissolved in dimethyl sulfoxide (Invitrogen) for 15 min. After washing with PBS three times, images were captured using a Revolve fluorescence microscope (Echo, San Diego, United States).

### RNA Extraction and Construction of RNA-seq Libraries for circRNAs and mRNAs

To characterize and express circRNAs during adipocyte differentiation in chickens, adipocytes were subjected to RNA-seq after 0, 12, 48, 72, and 120 h (*n* = 3 each group). Total RNA was extracted using TRIzol® reagent (Ambion, Austin, TX, United States) following the manufacturer’s instructions. RNA concentration and purity were assessed by monitoring OD260/280 nm and OD260/230 nm using a NanoDrop 2000 Spectrophotometer (Thermo Fisher Scientific, Wilmington, DE, United States). RNA integrity was detected using 1% Tris Acetate-EDTA (TAE) denaturing agarose gel electrophoresis and an Agilent Bioanalyzer 2100 system (Agilent Technologies, CA, United States). RNA samples with ratios of 28S/18S, band brightness of more than 1.5, RNA integrity number ranging from 8.0 to 10.0, OD260/280 nm of 1.8–2.0, and OD260/230 nm of 2.0–2.3 were qualified for RNA-seq.

Ribo-Zero RNA-seq libraries were prepared using the Ribo-Zero rRNA Removal Kit (Epicenter, Madison, WI, United States) and NEBNextR Ultra™ Directional RNA Library Prep Kit for IlluminaR (New England Biolabs, Ipswich, MA, United States) with 3 μg of RNA from each library according to the manufacturer’s recommendations. Briefly, total RNA samples were first treated with DNase I to remove genomic DNA pollution and with Ribo-Zero Gold Kits to remove rRNA. Following RNA fragmentation, the first strand of cDNA was synthesized with random hexamers using the RNA fragments as templates, and the second strand of cDNA was synthesized by adding a buffer, dNTPs, RNase H, and DNA Polymerase I. After purification with the QiaQuick PCR kit and elution with EB buffer, the fragments underwent terminal repair, poly-adenylation, adapter ligation, and agarose gel electrophoresis before PCR amplification. Constructed libraries were assessed using the Agilent Bioanalyzer 2100 system and then sequenced on the Illumina Novaseq 6000 platform following the 150 bp pair-end sequencing strategy.

### Construction of miRNA Libraries

For small RNA-sequencing, sRNA library construction was performed using NEBNext® Multiplex Small RNA Library Prep Set for Illumina® (New England Biolabs) with 3 μg of RNA from each library according to the manufacturer’s instructions. Briefly, the 3′-R adaptor was ligated at the 3′ end of the sRNA using T4 RNA ligase. The SR reverse transcription primer was appended to the free end of the 3′ SR adaptor that remains free after the 3′ ligation reaction and to hybridize transform the single-stranded DNA adaptor into a double-stranded DNA molecule. Then, the 5′-SR adaptor was ligated. Subsequently, the sgRNA with adapters was reverse-transcribed into cDNA. After PCR amplification, the library quality was evaluated on a bioanalyzer using a DNA 1000 chip according to the manufacturer’s instructions. Thereafter, the size selection of the libraries, referring to the bands corresponding to ∼140 bp for miRNAs, was performed using a 6% polyacrylamide gel electrophoresis. Finally, the purified products were subjected to small RNA-sequencing using the Illumina Novaseq 6000 platform.

### Identification and Differential Expression Analysis of circRNAs and mRNAs

Quality control of raw reads was carried out using in-house Perl scripts. The Q20, Q30, and GC content, as well as sequence duplication level, of the clean data were calculated. Raw data were filtered out with the removal of adapters, low-quality reads, and over 10% poly-N to yield clean reads. The clean reads were aligned to the chicken Galgal 6 reference genome using Burrows-Wheeler Aligner (BWA) to generate mapped reads (Li, 2013). Unmapped reads were processed for novel circRNA prediction using CIRI (Gao et al., 2015) and find_circ (Memczak et al., 2013). The expression of circRNA isoforms was normalized to spliced reads per billion mapped reads (SRPBM) based on the counts of circRNA junction reads (Arcinas et al., 2019). Differential analysis of circRNA expression levels was conducted using DESeq2 (Love et al., 2014). CircRNAs with a fold change of ≥1.5, and a *p*-value of < 0.05, were identified as differentially expressed.

To calculate the expression of protein-coding genes, mapped reads were generated from clean reads aligned to the chicken Galgal 6 genome using HiSAT2 (Kim et al., 2015). We then used the Stringtie software (Pertea et al., 2015) to assemble reads into transcripts and quantify gene expression normalized by fragments per kilobase of transcript per million fragments mapped (FPKM). The originally unannotated transcriptional regions were also located to identify novel genes, based on the comparison of assembled reads with the reference genome using Stringtie (Pertea et al., 2015). The expression of novel genes was also represented in FPKM. Furthermore, transcriptional factors in chickens were projected and classified using BLAST with AnimalTFDB (http://bioinfo.life.hust.edu.cn/AnimalTFDB/ (Hu et al., 2019) and Pfam databases (El-Gebali et al., 2019). Pairwise differential expression analysis was conducted using the DESeq2 (Love et al., 2014). Genes with |log2fold changes| ≥ 1 and false-positive discovery (FDR) < 0.01 were identified as differentially expressed genes (DEGs).

The DE-circRNAs and DEGs were subjected to K-means clustering using the Euclidean distance method with default parameters on the BMKCloud platform (https://www.biocloud.net/).

### Identification and Differential Expression Analysis of miRNAs

The clean reads were obtained using in-house Perl scripts after removing adaptor sequences, low-quality reads, over 10% poly-N, adapters, reads shorter than 18 or longer than 30 nucleotides (nt) of raw reads. The clean reads were matched with the chicken Galgal 6 genome assembly using Bowtie software (Langmead et al., 2009) against Silva, GtRNAdb, Rfam, and Repbase databases. rRNAs, transport RNAs (tRNAs), small nuclear RNAs (snRNAs), small nucleolar RNAs (snoRNAs), other ncRNAs, and repeat sequences were filtered out to obtain unannotated reads containing miRNAs. The mapped reads were generated *via* sequence alignment of the unannotated reads against the chicken Galgal 6 reference genome using Bowtie software. To identify known miRNAs, the mapped reads were aligned to known mature miRNAs together with their 2 nt upstream and 5 nt downstream sequences in miRbase (http://www.mirbase.org/index.shtml), allowing the maximum number of mismatched bases to be 1. Additionally, miRDeep2 was used to predict novel miRNAs. MiRNA expression levels in each sample were normalized using transcripts per million (TPM). Differential analyses of miRNA expression were performed using DESeq2 (Love et al., 2014). Differentially expressed miRNAs were identified with a fold change of ≥1.5 and a *p*-value of < 0.05.

### Functional Enrichment Analysis of circRNA-Parental Genes

GO and Kyoto Encyclopedia of Genes and Genomes (KEGG) pathway enrichment analyses of parental genes of DE-circRNAs were performed using the R package clusterProfiler (Yu et al., 2012). The COG database was used for orthologous classification of circRNA-parental genes (Tatusov et al., 2000). A *p*-value of < 0.05 was used as the cut-off value for KEGG pathway enrichment analysis.

### WGCNA of circRNAs and mRNAs

To describe the correlation patterns among circRNAs and mRNAs and determine the potential function of circRNAs that are involved in adipogenesis, a co-expression network was constructed using the R package WGCNA, based on all identified circRNAs and mRNAs (Langfelder and Horvath, 2008). First, we calculated a series of soft-thresholding power (from 1 to 30) according to the criterion of approximate scale-free topology and chose a soft-thresholding power value of 12 to analyze the network topology. The network was constructed using a signed type of topological overlap matrix (TOM), a minimal module size of 30, and a dendrogram cut height for module merging of 0.25. Then, module clustering analysis based on eigengenes was performed to explore the correlations among modules. Different adipocyte differentiation stages were used as trait files to evaluate the association of differentiation stages with the eigengene of each module. More attention was paid to the modules possessing extremely significant correlations with traits (*p* < 0.01) to compute the association of GS, which represents the correlation between genes and traits, with MM representing the correlation of the module eigengene and the gene expression profile. We focused on the modules satisfying the highly significant correlation (*p* < 0.01) between GS and MM, together with a significant correlation (*p* < 0.01) with a specific differentiation stage. CircRNAs and mRNAs with |GS| >0.8 and |MM| >0.8 were considered as hub genes in the corresponding modules. For such a module, the top 200 connections, ranked according to weight, were visualized using Cytoscape 3.8.2 (Shannon et al., 2003).

### Correlation of circRNA and Parental Gene Expression

To investigate the expression correlation of exonic, intronic, and intergenic circRNAs and corresponding parental protein-coding genes, we first retrieved the expression matrix of circRNA-parental gene pairs. Pearson correlation coefficients (PCCs) and *p* values of pairwise expression between circRNAs and their corresponding parental genes were computed. The circRNA-parental gene pairwise expression with a *p*-value < 0.05 was considered significant.

### Prediction of miRNA Targets of circRNAs and ceRNA Network Construction

The circRNA-miRNA-mRNA ceRNA network was constructed based on DE-circRNAs, DE-miRNAs, and DE-mRNAs. The interaction pairs of circRNA-miRNA and miRNA-mRNA were predicted using miRanda (http://www.microrna.org) (Betel et al., 2008) and TargetScan (http://www.targetscan.org/vert_72/) (Lewis et al., 2003). PCCs between matching circRNA-miRNA, miRNA-mRNA, and circRNA-mRNA pairs were computed based on their expression data with *p* values < 0.05. For a given positively co-expressed circRNA-mRNA pair, mRNAs and circRNAs that had a common MRE to interact with miRNAs and were negatively co-expressed with these miRNAs were feasible. The ceRNA network depicting circRNA-miRNA-mRNA was constructed using Cytoscape 3.8.2 (Shannon et al., 2003).

### Prediction of circRNA Translation

ORFs with a 150 nt minimum length of circRNAs were extracted using the online software getorf (http://emboss.sourceforge.net/apps/cvs/emboss/apps/getorf.html). The IRES of circRNAs was monitored via IRESfinder, a tool for IRES prediction in eukaryotic cells using framed k-mer features (Zhao et al., 2018).

### circRNA Validation and qRT-PCR Analysis

Exactly 1 μg of total RNA for each sample was reverse transcribed into cDNA using a FastKing RT Kit (with gDNase) (TIANGEN, Beijing, China) according to the manufacturer’s instructions. Genomic DNA (gDNA) was extracted *via* digestion with 10 μL of proteinase K (10 mg/ml) (Sigma) at a final concentration of 0.5% sodium dodecyl sulfate (Invitrogen). To validate circRNAs, we designed divergent primers for PCR amplification of the putative BSJ using cDNA as a template, and gDNA was used as a negative control. After electrophoresis on a 1% TAE agarose gel, the PCR products were confirmed via Sanger capillary sequencing (SinoGenoMax, Beijing, China).

To verify the gene expression of circRNAs and mRNAs in adipocytes at different differentiation stages, SYBR green-based qRT-PCR was performed in triplicate on a BioRadCFX96 Real-Time PCR system (BioRad, United States) in a 20 μL of total reaction volume containing 10 μL of 2 × SuperReal PreMix Plus (SYBR Green) (TIANGEN), 8 μL of RNase-free water, 0.5 μL of each forward and reverse primer (10 μM), and 1 μL of cDNA (approximately 300 ng). qRT-PCR amplification comprised an initial denaturation at 95°C for 15 min, 40 cycles of denaturation at 95°C for 10 s, annealing at an optimum temperature for 20 s, and extension at 72°C for 30 s, as well as a melting/dissociation curve stage. *GAPDH* served as an internal control to normalize the relative gene expression. The relative expression of genes was calculated using the 2−∆∆Ct method. The primers were designed using online National Center for Biotechnology Information (NCBI) Primer-BLAST (https://www.ncbi.nlm.nih.gov/tools/primer-blast/) and synthesized by SinoGenoMax (Supplementary Table S1).

### Statistical Analysis

All data are presented as the mean ± standard deviation (SD), and statistical significance was determined using T-test for differences between two group and one-way ANOVA for differences between three or more experimental groups by SPSS (version 25.0; IBM, Chicago, IL, United States) with different levels of significance: *p-value ≤ 0.05 and extreme significance **p-value ≤ 0.01. Graphics were produced using GraphPad Prism 8 (GraphPad Software, San Diego, CA, United States).

## Results

### Adipogenesis at Different Adipogenic Differentiation Stages of Chicken Abdominal Preadipocytes

**FIGURE 1:**
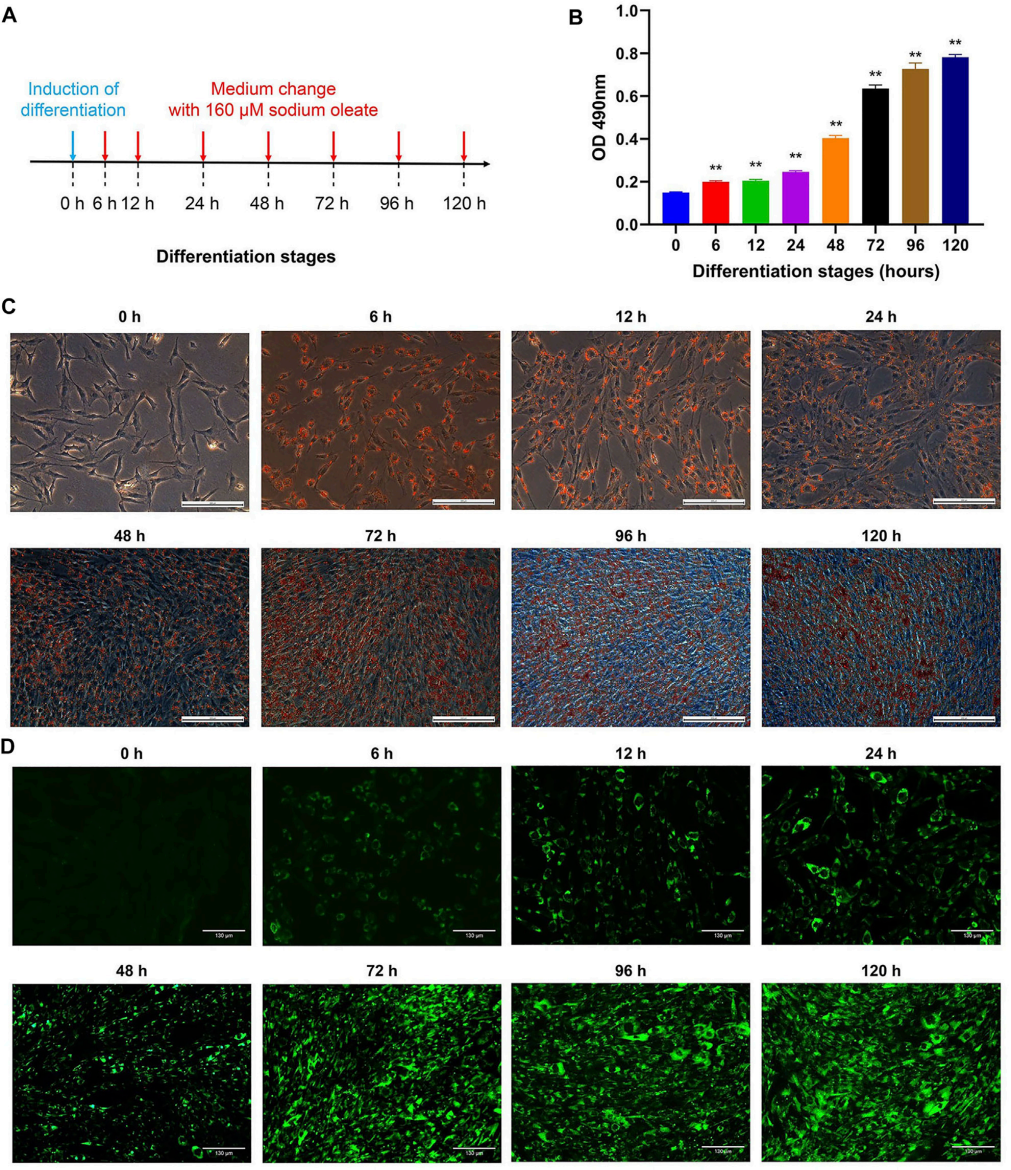
Adipogenesis of chicken abdominal preadipocytes at different differentiation stages. **(A)** Procedure for inducing adipogenic differentiation of abdominal preadipocytes in chicken; **(B)** Spectrophotometric analysis at the 490 nm absorbance of adipocytic lipid droplet stained by oil red O; **(C)** Microscopy of oil Red O staining of chicken abdominal adipocytes at 0, 6, 12, 24, 48, 72, 96 and 120 h after differentiation (20X); **(D)** BODIPY fluorescent staining of chicken abdominal adipocytes at 0, 6, 12, 24, 48, 72, 96 and 120 h after differentiation (20X).

The differentiation of chicken abdominal preadipocytes with sodium oleate was observed after 0, 6, 12, 24, 48, 72, 96, and 120 h of treatment (Figure 1A). Oil red O staining indicated that intracellular lipid droplets were first detected at 6 h after the induction of adipogenic differentiation, and their number gradually increased in a time-dependent manner until reaching a peak at 120 h (Figures 1B,C, Supplementary Figure S1). There was no significant difference in the accumulation of lipid droplets between 6 and 12 h, each of which showed a minor difference compared to that of 24 h. Moreover, gentle increase in the accumulation of lipid droplets was observed between 72 and 96 h, as well as 96 and 120 h. Similarly, BODIPY fluorescent staining in chicken abdominal preadipocytes also exhibited gradually increased lipid droplet accumulation (Figure 1D). These results suggested that the abdominal adipogenic differentiation models were successfully constructed in chickens.

### Identification of circRNAs in Chicken Abdominal Adipocytes

After inducing adipogenesis, the abdominal adipocytes incubated for 0, 12, 48, 72, and 120 h (*n* = 3 each group) were selected for RNA-seq. A total of 15 ribosomal RNA (rRNA)-depleted libraries, named A0 (A0-1, A0-2, A0-3), A12 (A12-1, A12-2, A12-3), A48 (A48-1, A48-2, A48-3), A72 (A72-1, A72-2, A72-3), and A120 (A120-1, A120-2, A120-3), were constructed and sequenced. A total of 253.30 G clean reads were obtained, and the percentage of clean reads with a Phred quality value over 30 ranged from 93.79 to 94.53%. An average GC content of 46.26% was detected in 15 samples. By matching the clean reads with the chicken reference genome Galgal 6, we found that the unique mapping ratios of 15 samples ranged from 87.03 to 92.64% (Table 1).

**TABLE 1:** Characteristics of the reads from 15 chicken adipocyte libraries.

Samples	Clean_Base (Gb)	Clean reads	Mapped reads	Unique map reads	GC (%)	Q20 (%)	Q30 (%)
A0-1	16.51	111,387,804	108,051,676 (97.00%)	96,943,816 (87.03%)	45.88	97.96	94.34
A0-2	16.61	112,000,780	108,910,392 (97.24%)	97,863,546 (87.38%)	45.7	97.68	93.8
A0-3	16.75	112,589,572	110,609,022 (98.24%)	100,552,088 (89.31%)	46.04	97.76	93.93
A12-1	16.61	111,756,856	110,089,558 (98.51%)	101,686,398 (90.99%)	46.27	97.97	94.37
A12-2	17.14	115,495,834	113,230,178 (98.04%)	103,090,257 (89.26%)	46.64	97.92	94.23
A12-3	16.38	110,348,698	108,489,036 (98.31%)	99,877,475 (90.51%)	46.31	98	94.45
A48-1	16.09	107,944,284	106,947,866 (99.08%)	99,994,308 (92.64%)	46.46	97.87	94.22
A48-2	19.38	130,001,758	128,719,618 (99.01%)	119,590,397 (91.99%)	46.22	97.69	94.03
A48-3	16.43	110,257,304	109,134,006 (98.98%)	101,894,550 (92.42%)	46.16	97.91	94.32
A72-1	16.96	113,843,732	112,446,252 (98.77%)	104,044,056 (91.39%)	46.04	97.9	94.27
A72-2	17.56	117,850,306	116,642,792 (98.98%)	108,265,039 (91.87%)	46.35	97.66	93.79
A72-3	16.30	109,961,180	107,253,706 (97.54%)	96,503,806 (87.76%)	46.15	97.54	93.79
A120-1	16.64	111,668,700	109,481,530 (98.04%)	99,241,207 (88.87%)	46.81	97.78	94.01
A120-2	17.98	120,868,414	118,350,068 (97.92%)	107,292,896 (88.77%)	46.63	97.94	94.3
A120-3	15.96	106,992,530	105,026,804 (98.16%)	96,154,578 (89.87%)	46.26	98.03	94.53

**FIGURE 2:**
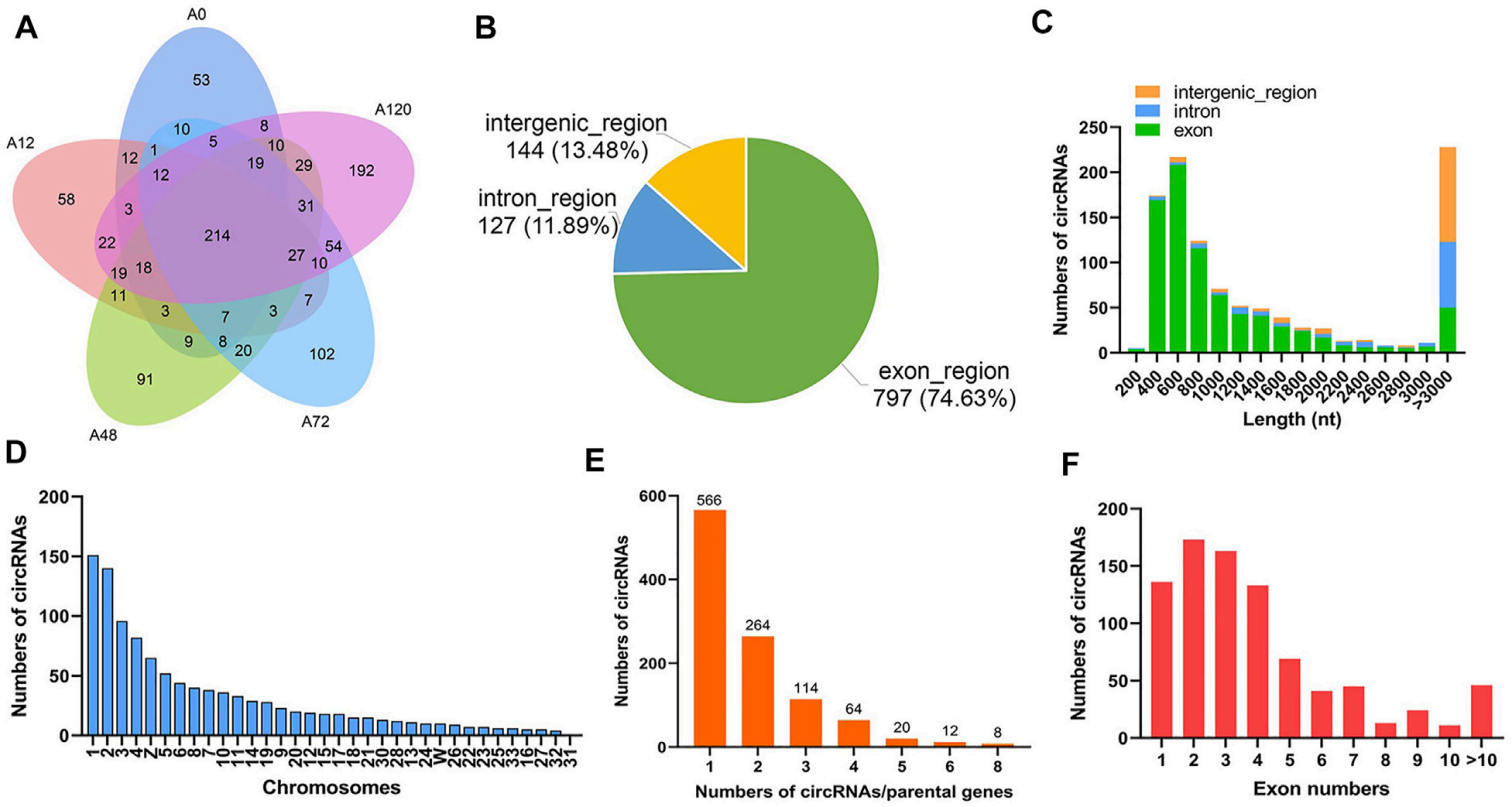
Characteristics of circRNAs in chicken abdominal adipocytes during differentiation stages. **(A)** Venn analysis of adipocyte circRNAs in each differentiation stage; **(B)** Distribution of genomic regions from where the identified circRNAs were derived; **(C)** Length distribution of the identified circRNAs; **(D)** Chromosomal distribution of the identified circRNAs; **(E)** The numbers of circRNAs derived from per parental gene; **(F)** Exon numbers of the identified circRNAs.

A total of 1,068 circRNAs, generated by back splicing from 755 linear mRNAs (55 novel genes and 700 unigenes including 61 transcription factors), were identified in 15 libraries and presented stage-specific expression in chicken abdominal adipocytes, incorporating 392 circRNAs in A0, 427 circRNAs in A12, 519 circRNAs in A48, 530 circRNAs in A72, and 673 circRNAs in A120. Of these, 53, 58, 91, 102, and 192 were specifically expressed in A0, A12, A48, A72, and A120, respectively (Figure 2A, Supplementary Table S2). The circRNAs consisted of 797 (74.63%) exonic circRNAs, 127 (11.89%) intronic circRNAs, and 144 (13.48%) intergenic circRNAs (Figure 2B), indicating that the majority of circRNAs consisted of protein-coding exons. The majority of circRNAs were characterized with a length of 400–800 nucleotides (nt) and more than 3,000 nt (Figure 2C). The top five chromosomes possessing the maximum numbers of circRNAs were Chr 1, Chr 2, Chr 3, Chr 4, and Chr Z (Figure 2D). Among the circRNA-parental gene pairs, 566 (53%) were derived from a single parental gene; in turn, the parental gene preferred to generate a single circRNA (Figure 2E). Most circRNAs were equipped with 1-4 exons (Figure 2F).

### Differentially Expressed circRNAs (DE-circRNAs) in Different Stages of Abdominal Preadipocyte Differentiation

**FIGURE 3:**
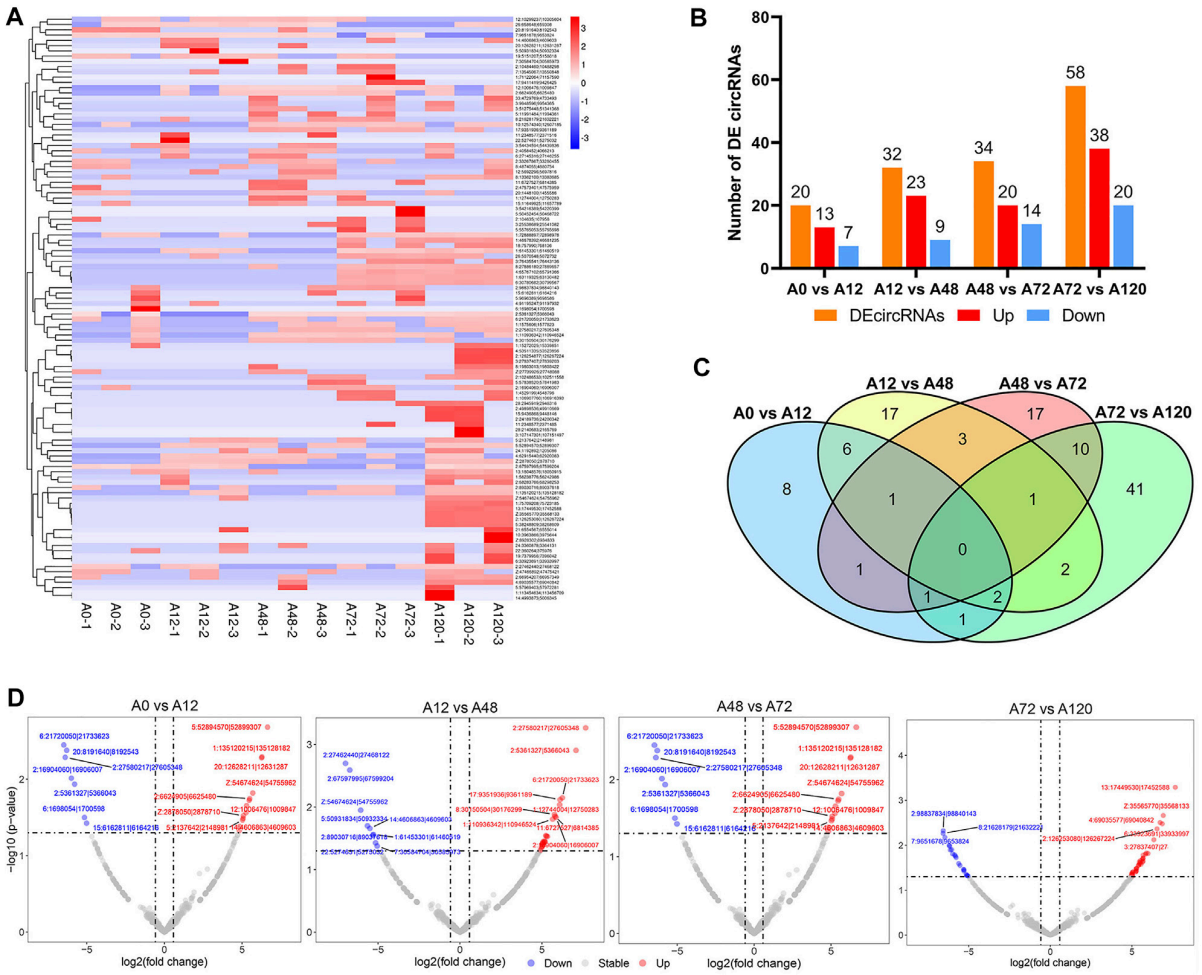
Differentially expressed circRNAs during chicken abdominal adipocytes differentiation stages. **(A)** Heatmap of differentially expressed circRNAs in four comparisons (A0 vs A12, A12 vs A48, A48 vs A72, A72 vs A120); **(B)** Histogram of four comparisons of the number of differentially expressed circRNAs; **(C)** Venn analysis of differentially expressed circRNAs; **(D)** Volcano map of differentially expressed circRNAs in A0 vs A12, A12 vs A48, A48 vs A72, A72 vs A120. The DE-circRNA with *p* value < 0.05 and log2foldchange > 5 were marked in red, and DE-circRNA with *p* value < 0.05 and log2foldchange < −5 were marked in blue.

By pairwise comparisons (A0 vs A12, A12 vs A48, A48 vs A72, A72 vs A120), a total of 111 DE-circRNAs were identified among adipocytes at different differentiation stages, and the number of DE-circRNAs gradually increased as the adipogenic differentiation of preadipocytes proceeded (Figure 3A, Supplementary Table S3). The number of significantly upregulated circRNAs was higher than that of significantly downregulated circRNAs in the four comparison groups (Figures 3B,D). No common DE-circRNAs were shared in the four comparison groups, and 8, 17, 17, and 41 DE-circRNAs were specific in A0 vs A12, A12 vs A48, A48 vs A72, A72 vs A120, respectively (Figure 3C).

### Validation of Candidate circRNAs

**FIGURE 4:**
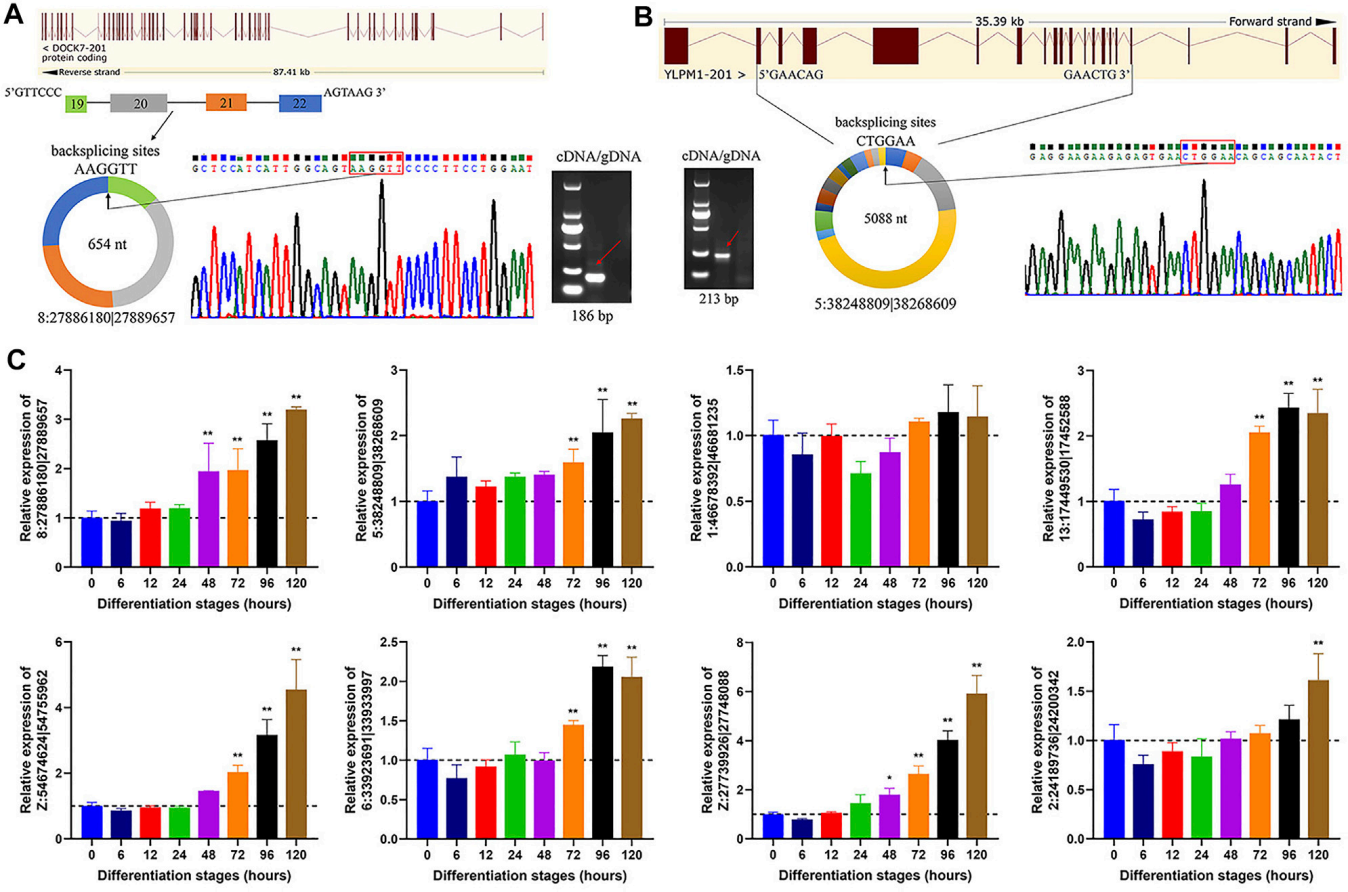
Cyclization and expression validation of circRNAs. **(A)** An example of circRNA validation. 8:27886180|27889657, derived from exon 19 to exon 22 of *DOCK7* gene, was indeed amplified with divergent primers in the cDNA but not gDNA form. Sanger sequencing confirmed the BSJ sites of 8:27886180|27889657. **(B)** An example of circRNA validation. 5:38248809|38268609, derived from exon 2 to exon 17 of *YLPM1* gene, was also indeed amplified with divergent primers in the cDNA but not gDNA form. Sanger sequencing confirmed the BSJ sites of 5:38248809|38268609. **(C)** Detection of circRNA expression in adipocytes at different differentiation stages using qRT-PCR. All circRNA expression values are shown as fold change values vs that of 0 h group. All data are represented as the mean ± SD (*n* = 3). *p < 0.05, **p < 0.01.

To confirm the authenticity of circRNAs candidates identified from transcriptome data, we randomly selected 8 circRNAs for experimental validation with divergent primers in both cDNA and gDNA templates, using PCR analysis coupled with Sanger sequencing. Using cDNA as template, the divergent primers were utilized to amplify back splicing sites of circRNAs, while gDNA should be expectedly negative without any amplification. A case in point that 8:27886180|27889657, an exonic circRNA with 654 nt in length, was produced by backsplicing that covalently linked the 3′ end of downstream exon 22 (splice donor) to 5′ end of upstream exon 19 (splice acceptor) of dedicator of cytokinesis 7 (*DOCK7*) gene (Figure 4A). Another exonic circRNA, 5:38248809|38268609, shared a length of 5,088 nt and was generated by covalently linking of exon 17 to exon 2 during transcriptional splicing process of YLP motif containing 1 (*YLPM1*) gene (Figure 4B). Additionally, the backsplicing sites of the 6 circRNA candidates were also indeed confirmed (Supplementary Figure S2).

To validate the expression levels of circRNAs during adipocyte differentiation stages, we detected the dynamic expression profiles of the above 8 circRNAs by qRT-PCR. These circRNAs showed a gradually increased expression level along with the adipogenic differentiation, except that 1:46678392|46681235, similar tendency, albeit although not statistically significant, was observed (Figure 4C), suggesting that they might participate in abdominal adipogenic differentiation in chicken.

### Functional Annotation of all circRNAs and DE-circRNAs

**FIGURE 5:**
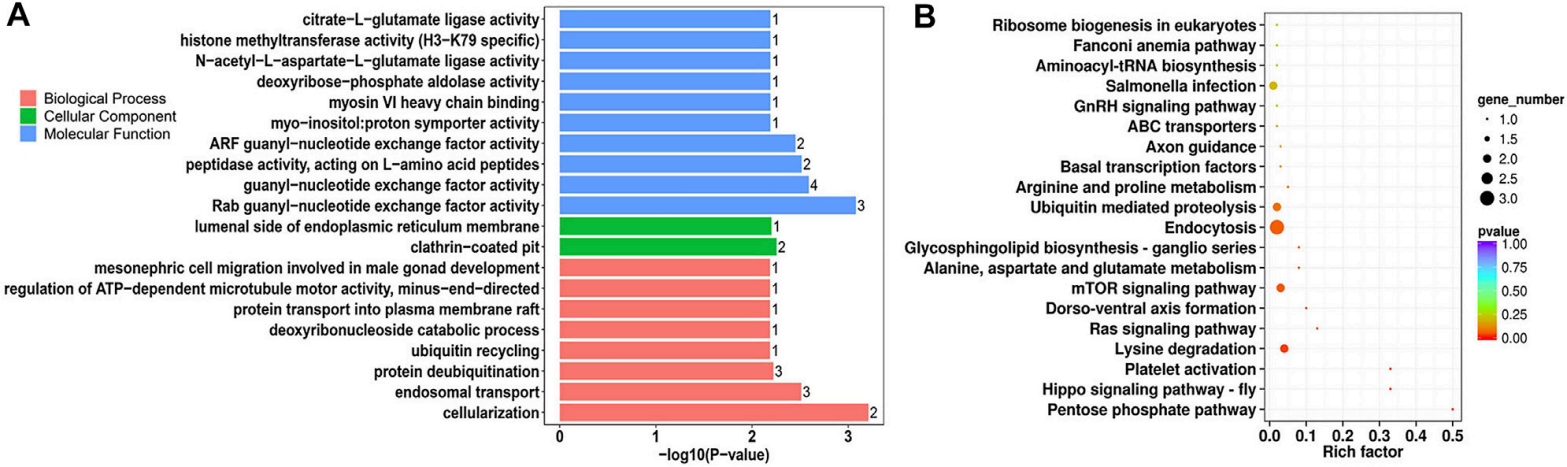
GO annotation and KEGG enrichment analysis of parental genes of DE-circRNAs. **(A)** Top 20 significantly enriched GO terms of DE-circRNAs in the biological process, cellular component and molecular function; **(B)** Top 20 KEGG pathways of DE-circRNAs.

Parental genes of all circRNAs were functionally enriched in 4,074 Gene ontology (GO) terms and 105 pathways (Supplementary Table S4). The top functions included zinc ion binding, insulin binding, centrosome, regulation of focal adhesion assembly, protein O-linked mannosylation (Supplementary Figure S3A), Wnt signaling pathway, apelin signaling pathway, ubiquitin-mediated proteolysis, and sphingolipid metabolism (Supplementary Figure S3B). The parental genes of DE-circRNAs were clustered into molecular binding-, enzymatic activity-, and development-related GO terms, which included Rab guanyl-nucleotide exchange factor activity, guanyl-nucleotide exchange factor activity, clathrin-coated pit, cellularization endosomal transport, and protein deubiquitination (Figure 5A, Supplementary Table S4). There were seven significantly enriched pathways for DE-circRNAs, including pentose phosphate, Hippo signaling, and mTOR signaling pathways (Figure 5B).

### Differentially Expressed miRNAs (DE-miRNAs) and Differentially Expressed Genes (DE-Genes) During Abdominal Preadipocyte Differentiation

In total, 1,458 miRNAs comprising 813 known miRNAs and 645 novel miRNAs were identified in 15 samples. Of these, 217 DE-miRNAs were tested among different differentiated adipocytes via four pairwise comparisons (A0 vs A12, A12 vs A48, A48 vs A72, A72 vs A120) (Supplementary Table S5). No common DE-miRNAs were found in the four comparison groups, and 35, 46, 40, and 38 DE-miRNAs were specific in A0 vs A12, A12 vs A48, A48 vs A72, A72 vs A120, respectively (Supplementary Figure S4A).

A total of 22,236 mRNAs, including 16,779 unigenes and 5,457 novel genes, were detected in 15 samples. Of these, 987 DE-genes were identified among different differentiated adipocytes *via* the above-mentioned four pairwise comparisons (Supplementary Table S6). Of these, 78, 269, 40, and 399 DE-genes were specific among these comparisons, respectively, and two DE-genes, ENSGALG00000047383 (WNT1 inducible signaling pathway protein 2, *WISP2*) and ENSGALG00000029606 (myosin, heavy chain 1E, *MYH1E*) were commonly differentially expressed among the four pairwise comparisons (Supplementary Figure S4B).

### Co-Expression Clustering Analysis of DE-circRNAs and DE-Genes

**FIGURE 6:**
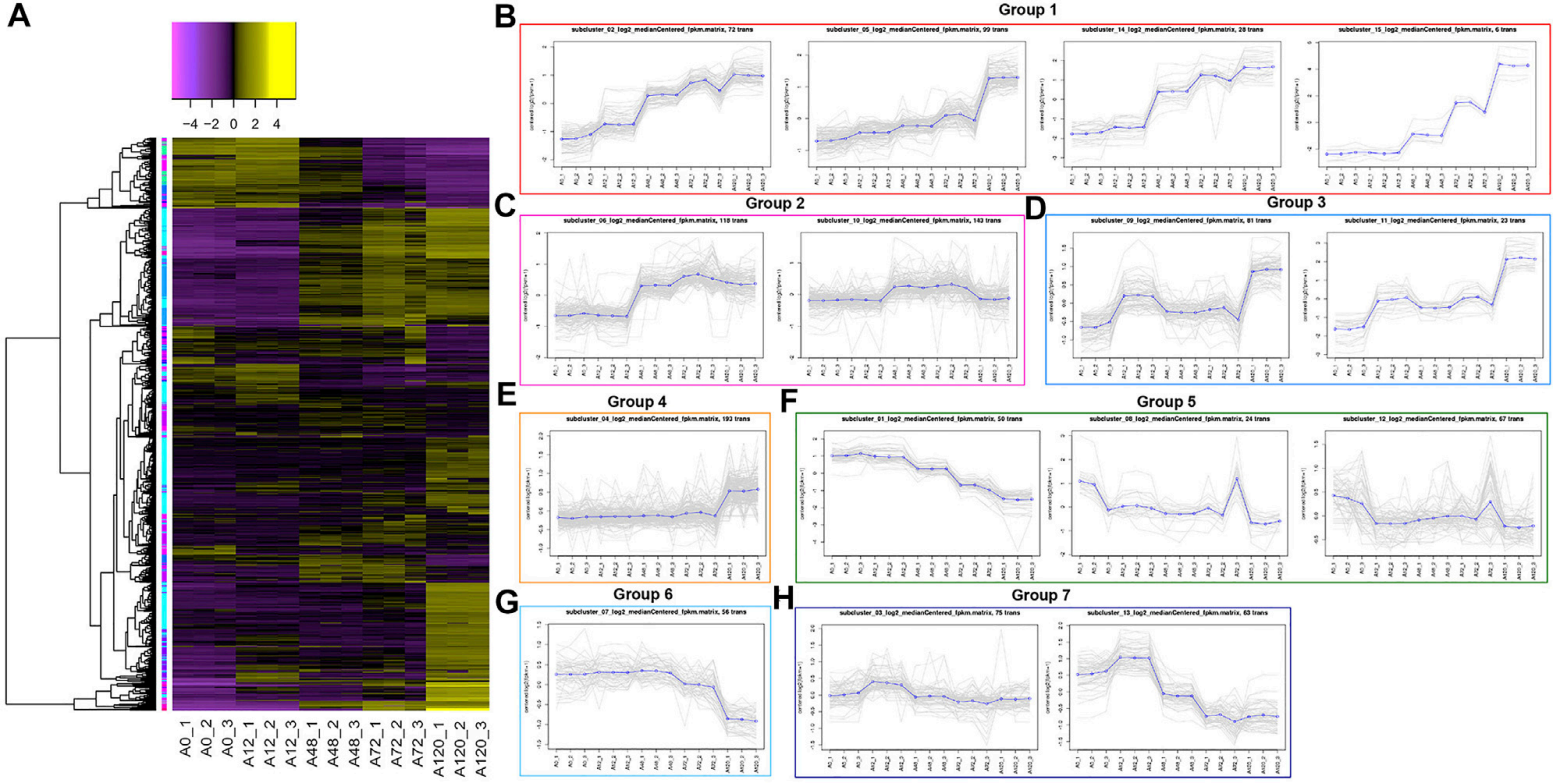
Clustering of all differentially expressed circRNAs and mRNAs. **(A)** Heatmap displays the K-means clustering of transformed expression abundances for circRNAs and mRNAs. Yellow means higher expression, and purple means lower expression; **(B)** Expression patterns of circRNAs and mRNAs in group 1 involving K02, K05, K14, K15 clusters; **(C)** Expression patterns of circRNAs and mRNAs in group 2 involving K06, K10 clusters; **(D)** Expression patterns of circRNAs and mRNAs in group 3 involving K09 and K11 clusters; **(E)** Expression patterns of circRNAs and mRNAs in group 4 involving K04 cluster; **(F)** Expression patterns of circRNAs and mRNAs in group 5 involving K01, K08, K12 clusters; **(G)** Expression patterns of circRNAs and mRNAs in group 6 involving K07 cluster; **(H)** Expression patterns of circRNAs and mRNAs in group 7 involving K03, K13 clusters.

To preliminarily explore the regulatory roles of circRNAs in chicken adipogenesis, we performed K-means clustering of all DE-circRNAs and mRNAs in 15 samples and revealed 15 clusters, which were further divided into seven groups according to dynamic expression (Figure 6A, Supplementary Table S7).

Group 1, including clusters 2, 5, 14, and 15, showed gradually enhanced expression levels of circRNAs and genes through all differentiation stages, including five circRNAs (1:46678392|46681235, 13:17449530|17452588, 2:102486533|102511558, 8:27886180|27889657, Z:35565770|35568133), as well as 194 genes, of which 11 were strongly associated with the lipid metabolism, such as *CPT1A*, *AADAC*, *ACSS2*, *ACSL1*, *AGPAT2*, *FABP4*, *CYP26B1*, and ENSGALG00000010433 (currently known as *FAAH*) (Figure 6B, Supplementary Table S7). Group 2, including clusters 6 and 10, showed circRNAs and genes whose expression levels increased after 48 and 72 h, but then decreased after 120 h, containing 28 circRNAs and 233 genes, of which five were lipid-related genes, such as *ABCA12*, *DEGS2*, and *CYP4V2* (Figure 6C, Supplementary Table S7). Group 3, including clusters 9 and 11, exhibited circRNAs and genes that were upregulated after 12 h, but were modestly downregulated after 48 h and then upregulated again after 72 and 120 h, peaking after 120 h. A lipid-related gene, *STARD5*, was detected in this group (Figure 6D, Supplementary Table S7). Group 4, including clusters 4, displayed a stable expression abundance from 0 to 72 h, whereas a dramatic increase after 120 h, including 50 circRNAs and 143 genes, such as lipid-related genes *ELOVL7*, *ABCA1*, *DDHD2*, and *Gallus_gallus_newGene_4376* (Figure 6E, Supplementary Table S7). Group 5, including clusters 1, 8, and 12, exhibited a repressed expression trend of circRNAs and genes after adipogenesis induction, including nine circRNAs and 132 mRNAs. Of these, *SC5D*, ENSGALG00000053860 (currently annotated as *mycocerosic acid synthase-like*), and ENSGALG00000048205 (also known as *EBP*) were related to the lipid metabolism (Figure 6F, Supplementary Table S7). CircRNAs and genes in group 6, referring to cluster 7, maintained steady expression until 48 h, which declined until 120 h when the lowest level was achieved, including two circRNAs, 12:10299237|10305604 and 7:9651678|9653824, in addition to 54 genes, among which *BBOX1* was lipid-related (Figure 6G, Supplementary Table S7). The expression of circRNAs and genes in group 7, including clusters 3 and 13, increased after 12 h and then decreased until 120 h. Ten circRNAs and 128 genes were detected, including 17 lipid-related genes, such as *STARD4*, *ACSBG2*, *AACS*, *DHCR7*, *SCD*, *FADS2*, *ACAT2*, *MVK*, *HMGCS1*, *HMGCR*, *SQLE*, and *ELOVL6* (Figure 6H, Supplementary Table S7).

### Weighted Gene Co-Expression Network Analysis of all circRNAs and mRNAs

**FIGURE 7:**
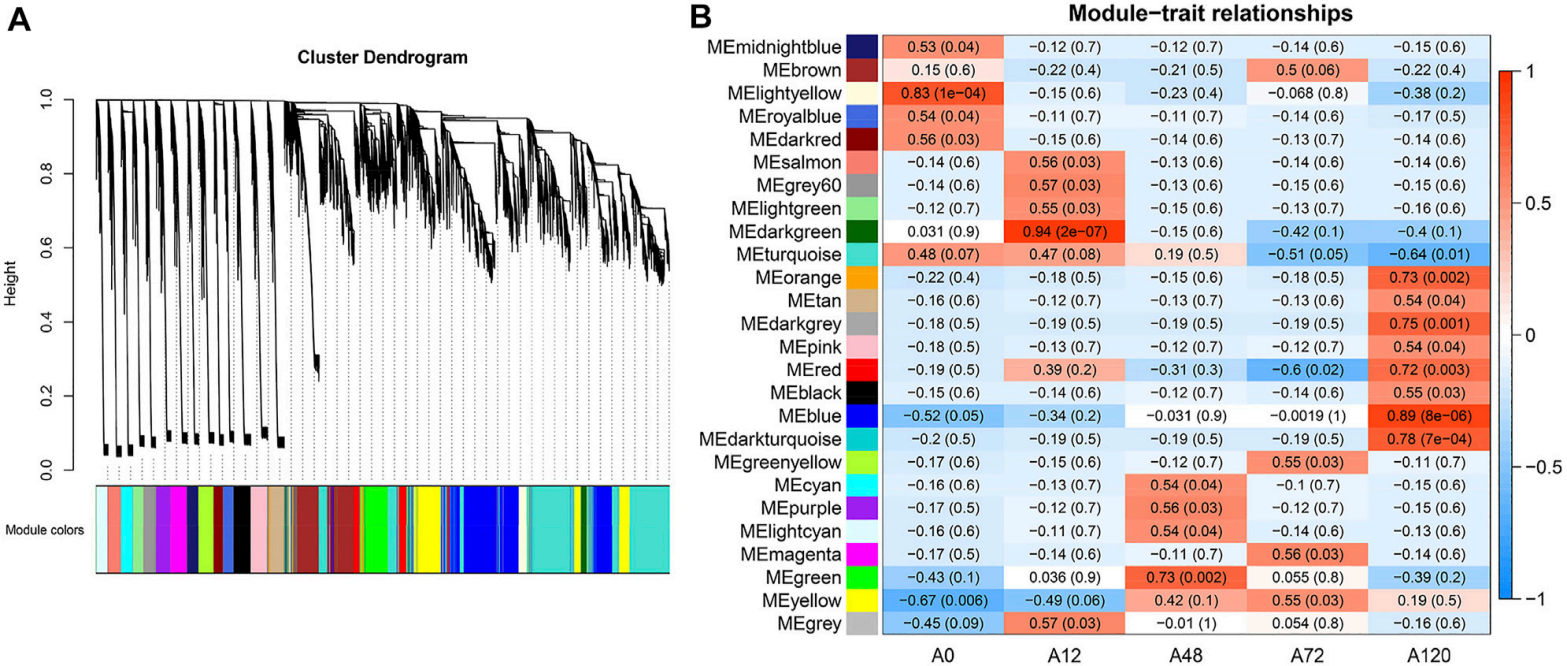
WGCNA of all circRNAs and mRNAs identified from chicken abdominal adipocytes over four differentiation stages. **(A)** Hierarchical cluster tree showing 26 modules of co-expressed genes. Each gene is represented by a leaf in the tree, and each module is represented by a major tree branch. The lower panel shows the modules in designated colors, in which module “grey” refers to unassigned genes; **(B)** Correlation analysis of module and differentiation stages. The color ranging from blue to red in heatmap indicates higher correlation.

We first combined the expression matrix of all circRNAs and mRNAs for WGCNA to identify modules of strongly co-expressed genes. Here, a soft-thresholding power value of 12 was chosen to analyze the network topology (Supplementary Figure S5). A total of 26 modules, including 21,227 filtered RNAs ranging in size from 42 RNAs (7 circRNAs and 35 genes) in the orange module to 4,804 RNAs (54 circRNAs and 4,750 genes) in the turquoise module were included (Figure 7A, Supplementary Table S8). As shown in Figure 7B, ten stage-specific modules were identified (*p* < 0.01), and the light yellow module was significantly positively correlated with A0, the dark green module was significantly positively correlated with A12, and the green module was significantly positively correlated with A48. The orange, dark grey, red, blue, and dark turquoise modules were significantly positively correlated with A120. Conversely, the yellow and turquoise modules were negatively correlated with A12 and A120, respectively. For each of the ten noteworthy modules, the enriched pathways of these protein-coding genes included lipid-related pathways such as PPAR signaling pathway, fatty acid metabolism, MAPK signaling pathway, adipocytokine signaling pathway, steroid biosynthesis, TGF-beta signaling pathway, ether lipid metabolism, NOD-like receptor signaling pathway, and ABC transporters (Supplementary Table S9).

**FIGURE 8:**
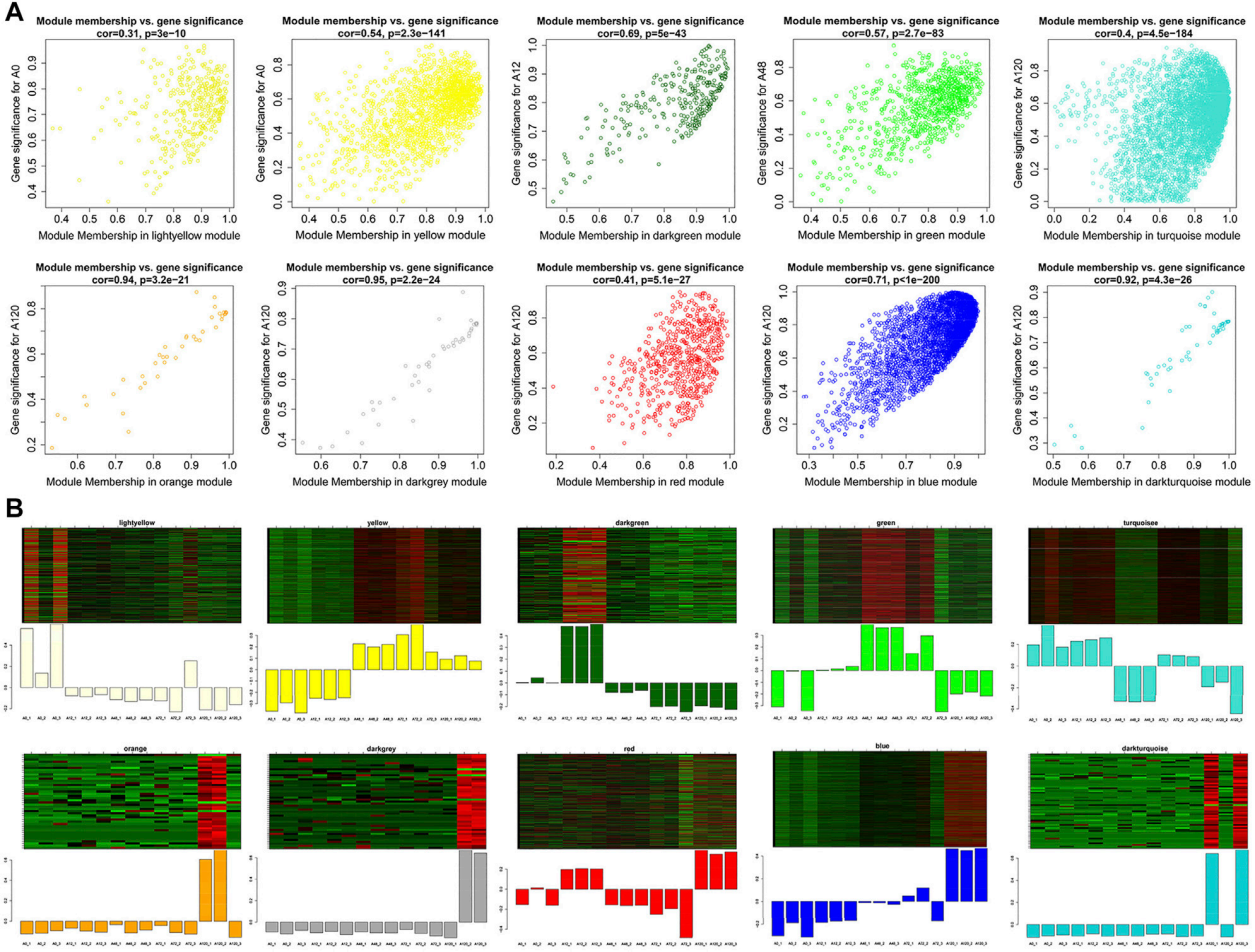
Visualization of GS vs MM and expression level in 10 interesting modules. **(A)** A scatterplot of GS for differentiation stage vs MM in modules; **(B)** The heatmap and bar plot represent the expression level of genes in modules in 15 samples. The color ranging from green to red in heatmap indicates higher expression levels.

Next, we identified genes that were highly significant at a certain stage with a high module membership in the above ten modules using gene significance (GS) and module membership (MM) measures. In terms of the ten modules that were significantly linked to differentiation stages, each showed a significantly high correlation between GS and MM (Figure 8A). Accordingly, the expression levels of genes in the light yellow module achieved an uppermost peak in A0, matching its significantly positive correlation with the light yellow module at the A0 stage (*P* = 1e-04). Likewise, the lowest expression levels of genes in A0 within the yellow module confirmed the negative correlation. The uppermost expression levels of genes in A12 within the dark green module and A48 within the green module conformed to their positive correlation. The orange, dark grey, red, blue, and dark turquoise modules exhibited the highest expression levels of genes in A120, which confirmed their positive correlation with A120 (Figure 8B). There were 219 circRNAs in the ten noteworthy modules, among which 58 circRNAs were differentially expressed (Supplementary Table S8). For each of the ten noteworthy modules, circRNAs with |GS| >0.8 and |MM| >0.8 were identified as potential hub genes, among which 12 and 1 hub circRNAs were found in blue and turquoise modules, respectively, and no other hub circRNAs were identified in the other modules, in which eight DE-circRNAs were identified (Supplementary Table S10).

Furthermore, to explore the network connections among the most connected genes in these modules, we visualized genes with the top 200 connectivity for each module using Cytoscape. Respectively, 5, 5, 3 and 9 circRNAs were found in orange, dark grey, blue, and dark turquoise models, which were considered as the most connected circRNAs (Supplementary Figure S6). Among these, four DE-circRNAs, 15:9436868|9448146, 2:24189736|24200342, 2:49898536|49910669, and 24:1192892|1205086, were in the top 200 for the orange module; four DE-circRNAs, 19:7379956|7396042, 2:68283766|68298253, 22:360264|375976, and 6:33923691|33933997, for the dark grey module; two DE-circRNAs, Z:35565770|35568133 and Z:54674624|54755962, for the blue module; three DE-circRNAs, 2:126254877|126267224, 3:27837407|27839203, and Z:27739926|27748088, for the dark turquoise module (Supplementary Table S8). Of these, Z:35565770|35568133 and Z:54674624|54755962 were identified as hub genes and were also differentially expressed, suggesting their crucial roles in chicken adipogenic differentiation, and were considered as candidate functional circRNAs.

### Correlation of Transcriptional Expression Between circRNAs and Their Parental Genes

**FIGURE 9:**
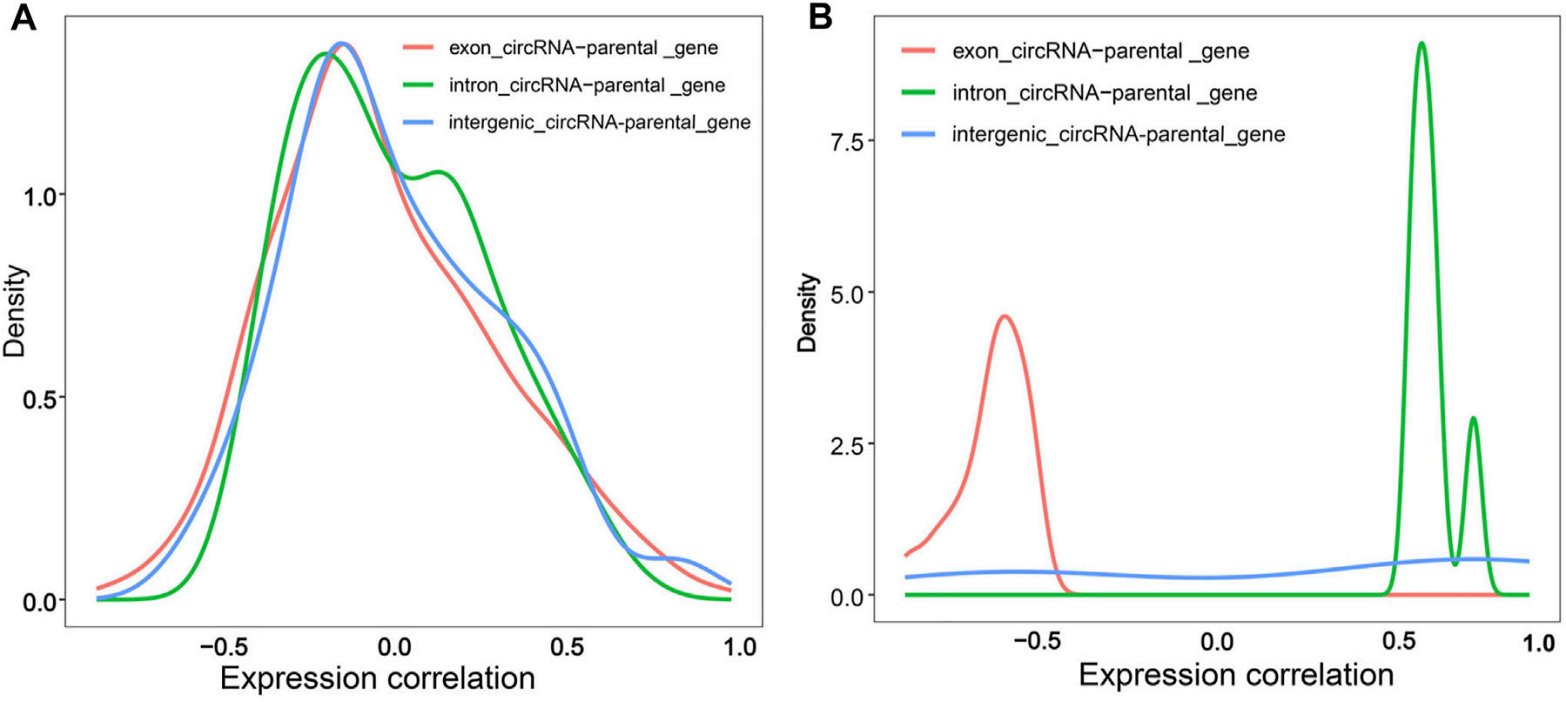
Density diagram of correlation between circRNAs and their parental genes. **(A)** Density diagram of correlation between circRNAs and their parental genes; **(B)** Density diagram of significant expression correlation between circRNAs and their parental genes (*p* < 0.05). The red, green and blue line represents the expression correlation of exonic circRNA-parental gene pairs, intronic circRNA-parental gene pairs and intergenic circRNA-parental gene pairs, respectively.

To confirm whether circRNAs affect parental gene expression, we first calculated pairwise expression correlation between exonic/intronic/intergenic circRNAs and their parental genes. Overall, the three types of circRNAs showed a diverse co-expression trend compared to their parental genes, especially for exonic circRNAs, and the majority were negatively correlated with their parental genes (rs < 0) (Figure 9A, Supplementary Table S11). Exactly 36 exonic circRNAs were considered as effective circRNA-parental gene pairs with a *p*-value of < 0.5, all of which exhibited a negative correlation with an rs of < −0.5. Moreover, six effective intronic circRNA-parental gene pairs were identified and consistently showed a positive correlation with an rs of >0.5. Eight effective intergenic circRNA-parental gene pairs were detected, including five that showed a positive correlation with an rs of >0.5 and three sharing a negative correlation with an rs of < −0.5 (Figure 9B, Supplementary Table S11). These results suggested that exonic circRNAs might inhibit the expression of their parental genes, in contrast to intronic circRNAs, which could enhance the expression of their parental genes. Moreover, intergenic circRNAs could inhibit or enhance the expression of their parental genes, although more in favor of enhanced expression. The expression of a candidate functional circRNA, Z:35565770|35568133, an exonic circRNA, exhibited a strong negative correlation with that of its parental gene, abhydrolase domain containing 17B, depalmitoylase (*ABHD17B*) (rs = −0.76, *p* < 0.001). The expression pattern of Z:54674624|54755962, an intergenic circRNA, was poorly correlated with that of its parental gene *ENSGALG00000015428* (rs = 0.37, *p* = 0.17).

### circRNA-miRNA-mRNA ceRNA Network

To elucidate the ceRNA regulatory mechanism underlying the roles of circRNAs during abdominal preadipocyte differentiation in chickens, we constructed a circRNA-miRNA-mRNA ceRNA regulatory network. A total of 1,204 circRNA-miRNA pairs including 530 miRNAs, with 70 DE-miRNAs, were predicted as potential targets of DE-circRNAs, wherein 46 DE-circRNAs interacted with more than three miRNAs, and Z:54674624|54755962 shared a maximum of 349 miRNAs (Supplementary Table S12). Of these, expression of two DE_circRNAs, Z:54674624|54755962 and 10:12574340|12607185, was significantly negatively correlated with that of five DE-miRNA, gga-miR-1635, miR-92-5p, novel_miR_232, novel_miR_263 and gga-miR-12226-5p. These five DE-miRNAs could negatively interact with 346 DE-mRNA targets to generate 411 DE-miRNA-DE-mRNA pairs. Additionally, 97 DE-mRNAs showed a significantly positive correlation with an identical pattern to the two DE-circRNAs, Z:54674624|54755962 and 10:12574340|12607185, to generate 129 DE-circRNA-DE-mRNA pairs.

**FIGURE 10:**
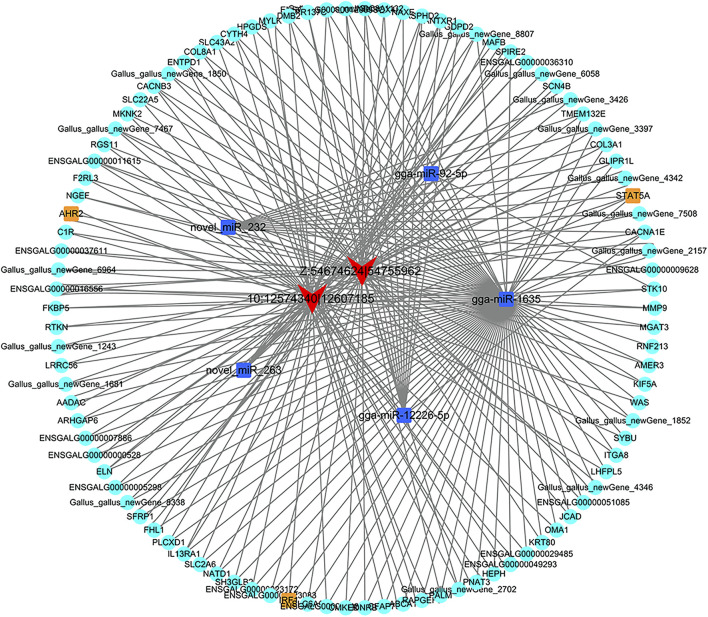
The ceRNA regulatory network of circRNA-miRNA-mRNA in chicken abdominal adipocytes from different differentiation stages. The triangle filled with red represents circRNAs, the rectangles filled with blue represents miRNAs, the circles filled with orange and turquoise represents transcription factor and genes, respectively.

Z:54674624|54755962 sponged two potential DE-miRNAs, gga-miR-1635 and novel_miR_232, wherein the activator of transcription 5A gene (*STAT5A*), the protein product of which functions as a transcriptional factor, was a potential target of novel_miR_232. Other two transcriptional factors, aryl hydrocarbon receptor 2 (AHR2) and interferon regulatory factor 1 (IRF1), were potentially targeted by gga-miR-1635, along with genes related to lipid metabolism, including signal transducer and mannosyl (beta-1,4-)-glycoprotein beta-1,4-N-acetylglucosaminyltransferase (*MGAT3*), arylacetamide deacetylase (*AADAC*), ATP-binding cassette, sub-family A (ABC1), and member 1 (*ABCA1*). Another circRNA, 10:12574340|12607185, could bind to three miRNAs, gga-miR-92-5p, gga-miR-12226-5p, and novel_miR_263, wherein *STAT5A* could also be targeted by novel_miR_263, and serine/threonine kinase 10 (*STK10*) could be targeted by gga-miR-92-5p (Figure 10).

### Potential Translation Capacity of circRNAs

**FIGURE 11:**
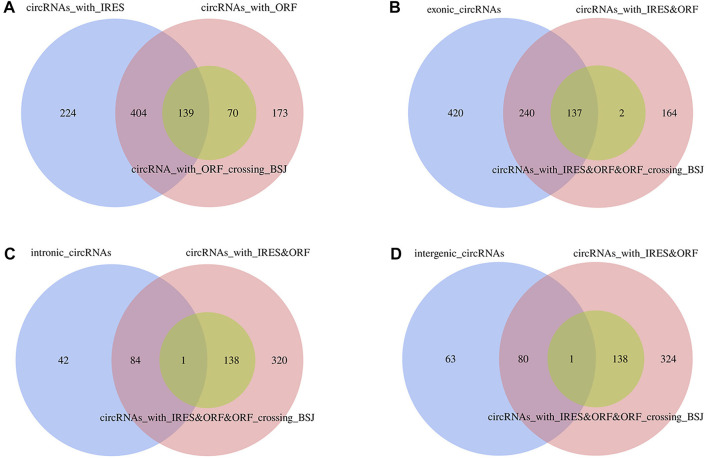
Translation capacity prediction of circRNAs based on ORF and IRES sequences. **(A)** Veen analysis of circRNAs with IRES, ORF and ORF crossing the backsplicing junctions; **(B)** Veen analysis of exonic circRNAs and circRNAs with IRES and ORF, circRNAs with IRES and ORF crossing the backsplicing junctions; **(C)** Veen analysis of intronic circRNAs and circRNAs with IRES and ORF, circRNAs with IRES and ORF crossing the backsplicing junctions; **(D)** Veen analysis of intergenic circRNAs and circRNAs with IRES and ORF, circRNAs with IRES and ORF crossing the backsplicing junctions.

Based on the elucidated point that endogenous circRNAs with open reading frames (ORFs) could be efficiently translated into proteins driven by the internal ribosome entry site (IRES), we wanted to identify whether adipocyte circRNAs have a protein-coding potential in chicken. We predicted ORF and IRES of 1,068 circRNA sequences. The results showed that a total of 786 circRNAs possessed ORFs, 209 of which were equipped with ORFs crossing the back-splicing junction. In addition, 767 circRNAs were identified by IRES. There were 543 circRNAs exhibiting both IRES and ORF, including 139 circRNAs with both IRES and ORFs crossing the back-splicing junction (Figure 11A, Supplementary Table S13). Limited to the circRNA types, 377 exonic circRNAs, 85 intronic circRNAs, and 81 intergenic circRNAs were overlapped among circRNAs satisfying IRES and ORFs, with overlapping corresponding 137 exonic circRNAs (Figure 11B), 1 intronic circRNA (Figure 11C), 1 intergenic circRNA (Figure 11D) with IRES and ORFs crossing the back-splicing junction, respectively. Both Z:35565770|35568133 and Z:54674624|54755962 were only equipped with IRES sequences, without ORFs, indicating their poor protein-encoding potential.

## Discussion

Obesity, which is characterized by excessive adiposity and the disruption of metabolic homeostasis in adipose tissue, has been an escalating health burden with increasing prevalence worldwide. CircRNAs are ubiquitous across all eukaryotic tissues, and most circRNAs are highly conserved among closely related species (Santos-Rodriguez et al., 2021). The pro-adipogenic function of circArhgap5-2 is conserved between human and mouse adipocytes (Arcinas et al., 2019). As commercial broilers have been utilized as a good biomedical model to study the basic mechanisms of adipogenesis, obesity, and obesity-related diseases (Abdalla et al., 2018), it is of great significance to study the formation and regulatory mechanisms underlying chicken abdominal fat deposition mediated by circRNAs, which could provide feasible breeding programs for animal production and facilitate the development of new therapeutic methods for obesity in humans. In this study, we successfully we induced adipogenesis from chicken abdominal preadipocytes and performed comprehensive profiling of circRNAs in chicken abdominal adipocytes from different differentiation stages using Ribo-Zero RNA-seq.

A total of 1,068 circRNAs were identified in abdominal adipocytes over five differentiation stages, which was different from those of chicken granulosa cells (11,642 circRNAs) (Shen et al., 2019), embryonic muscle (13,377 circRNAs) (Ouyang et al., 2018b), and spleen (2,169 circRNAs) (Wang L. et al., 2020), which might be a consequence of the tissue-specific expression of circRNAs or our more restrictive qualification standard for circRNAs (Salzman et al., 2013). Moreover, the number of identified circRNAs in A120 reached a peak and exhibited a gradual increase as chicken abdominal preadipocyte differentiation approached, suggesting that circRNAs were expressed in a stage-specific manner. This might be explained by the fact that circularization resulting from pre-mRNA back splicing is a tightly and precisely regulated event during abdominal preadipocyte development in chickens, thereby illustrating the diverse regulatory roles of circRNAs in chicken adipogenesis. Our study revealed that circRNAs shared a maximum number of two exons, following three exons, in chicken abdominal adipocytes, in agreement with a previous report which showed that most circRNAs comprised several exons, usually two or three (Zhang et al., 2014). To date, lariat-driven circularization, intron pairing-driven circularization, and RNA binding protein (RBP)-driven circularization for the biosynthesis of exonic, intronic, and intergenic circRNAs during pre-mRNA splicing have been proposed (Fan et al., 2017). Most of the circRNAs were derived from protein-coding exons. Consistent with this, 74.63% of exonic circRNAs were the most abundant circRNA type, accounting for over 70% of the identified circRNAs in chicken abdominal adipocytes. Generally, a single linear mRNA produces one circRNA, sometimes multiple circRNAs, owing to competition for RNA pairing across introns or diverse genomic origins (Chen, 2016). Our findings indicated that a single linear mRNA principally generated only one circRNA, and some could yield two or more circRNAs, up to eight, suggesting that a complicated circularization mechanism indeed occurred in circRNA biogenesis in chicken abdominal adipocytes.

Previous studies have demonstrated that circRNAs play crucial roles in lipid metabolism, including hepatocellular triglyceride accumulation and lipid peroxidation in humans (Guo et al., 2017; Guo et al., 2018), adipocyte proliferation and differentiation in mammals and cattle (Arcinas et al., 2019; Jiang et al., 2020), IMF deposition in yaks and donkeys (Li et al., 2020a; Wang H. et al., 2020), hepatic lipid biosynthesis in pigs (Huang et al., 2018), and subcutaneous fat deposition in pigs and Chinese buffalo (Li A. et al., 2018; Huang et al., 2019). Here, the parental genes of all identified circRNAs were mainly enriched in molecular binding, enzymatic activity, and development-related GO terms, indicating that chicken adipogenesis is a complex process that incorporates numerous events. In addition, several pathways related to lipid metabolism were significantly enriched, including the pentose phosphate pathway, Hippo signaling pathway, and mTOR signaling pathway, suggesting that circRNAs could indeed serve as regulator molecules to participate in abdominal preadipocyte differentiation in chicken.

Because of the generally considered non-coding property and limited functional studies on circRNAs, particularly in chicken, we employed WGCNA to construct a co-expression network of circRNAs and protein-coding genes and further inferred the potential roles of circRNAs in chicken abdominal preadipocyte differentiation. Of the 26 modules, ten were significantly correlated with the differentiation stages of abdominal preadipocytes and significantly enriched in pathways related to lipid metabolism in chickens, thereby reflecting their stage-specific relationships and importance during adipogenic differentiation. This implied that circRNAs within these ten modules were related to chicken adipogenesis. Combined with the K-means clustering analysis based on the DE-circRNAs, we identified a subset of circRNAs that were responsible for chicken abdominal preadipocyte differentiation progression, collectively revealing the regulatory roles of circRNAs in chicken adipogenesis. In particular, two circRNAs Z:35565770|35568133 and Z:54674624|54755962, which were identified as the most connected hub circRNAs and found in the blue module, significantly correlated with the late stage of preadipocyte differentiation via WGCNA. These were also differentially expressed and clustered in K05 and K11, respectively, suggesting their crucial roles in chicken adipogenic differentiation and function as candidate circRNAs related to abdominal fat deposition in chickens.

It has been demonstrated that as competition between the linear mRNA splicing and back splicing of exons occurrs during gene transcription, exonic circRNA could function in parental gene regulation by competing with linear splicing (Ashwal-Fluss et al., 2014). Intronic circRNAs modulate the transcription of their parental genes by interacting with RNA polymerase II (Pol II) (Zhang et al., 2013). Exon-intron circRNAs (EIciRNAs), which are predominantly localized in the nucleus, interact with U1 snRNP and thus promote the transcription of their parental genes (Li et al., 2015). Therefore, we detected the correlation of expression between circRNAs and parental genes and found that the expression of exonic circRNAs was negatively correlated with that of their parental genes, suggesting that exonic circRNAs could inhibit the transcription of their parental genes during chicken abdominal adipocyte differentiation, and vice versa, which might be attributable to their competition with linear splicing. Expression of intronic circRNAs exhibited a positive correlation with that of their parental genes, indicating that intronic circRNAs could stimulate the transcription activity of their parental genes, perhaps through interacting with RNA Pol II. Considering both inhibited and enhanced transcription activity, the regulatory roles of intergenic circRNAs may be more complex on their parental genes. Controversially, it has been argued that there is no relationship between the expression of circRNA and that of their linear mRNA isoform (Salzman et al., 2013), suggesting that circRNA expression could not be generally explained by a simple correlation with the expression of their linear isoforms, which involves a potentially novel, widespread, and intricate layer of gene regulation, and further studies are needed. Of these, expression of Z:35565770|35568133, which might function as a crucial mediator in chicken adipogenic differentiation, exhibited a significantly negative correlation with that of its parental gene *ABHD17B*. ABHD17B, a member of the ABHD protein family and a lipid-metabolizing enzyme at the interface of cell signaling and energy metabolism in mammals, plays a vital role in lipid metabolism, lipid signal transduction, and metabolic disease (Lord et al., 2013). Therefore, we hypothesized that Z:35565770|35568133 might compete with linear pre-mRNA splicing of *ABHD17B* gene to inhibit *ABHD17B* mRNA expression during adipogenic differentiation, which needs to be further verified.

Numerous studies have shown that circRNAs, which contain many miRNA response elements (MREs), can act as ceRNAs to sponge miRNAs, resulting in competitive elimination of their inhibition and subsequently playing regulatory roles in diseases and metabolic activities. miRNAs are a large class of endogenous non-coding RNAs that post-transcriptionally regulate gene expression and play important roles in adipocyte development and adipogenesis (Chen et al., 2013). It has been reported that downregulation of miR-92a expression could inhibit lipid accumulation induced by oxidized low-density lipoprotein in murine macrophage cells (RAW264.7) (Sun et al., 2011). Here, as a member of miR-92 family, gga-miR-92-5p exhibited gradually decreased expression as abdominal preadipocyte differentiation progressed, suggesting its regulatory roles in chicken adipogenesis. The circRNA, 10:12574340|12607185, potentially interacted with gga-miR-92-5p, which might target STK10, a member of the serine/threonine kinase family that phosphorylates and activates members of the AMPK-related sub-family of protein kinases and hence regulates lipid metabolism (Herms et al., 2015; Shan et al., 2015). 10:12574340|12607185 might sponge novel_miR_263 targeting STAT5A, which encodes a transcription factor that mediates signals for a broad spectrum of cytokines and was shown to participate in mammalian lipid accumulation and fatty acid metabolism and function as an initial mediator of adipogenesis (Nanbu-Wakao et al., 2002; Barclay et al., 2011; Dong et al., 2019; Kaltenecker et al., 2020). These findings suggest that 10:12574340|12607185 could sponge gga-miR-92-5p to alleviate its silencing on the targeted STK10 and/or novel_miR_263 to promote STST5A gene expression, thereby regulating abdominal adipogenic differentiation in chickens.

Additionally, *STAT5A* could act as another target of gga-miR-1635 and novel_miR_232, which potentially interacts with Z:54674624|54755962. Two other targets, AHR2, and IRF1, encode transcriptional factors for gga-miR-1635. AHR2 belongs to the AHR family, a sensor of small molecules including dietary components. It has been identified as a potential regulator of lipid homeostasis maintenance (Biljes et al., 2015). IRF1 functions as a mediator of adipocyte inflammatory phenotypes and alters lipid droplet composition of adipocytes, and its overexpression leads to insulin resistance and attenuated lipolysis (Friesen et al., 2017). Notably, MGAT3 belongs to the MGAT family of enzymes that catalyze the synthesis of diacylglycerol from monoacylglycerol, a committed step in triglyceride formation (Cao et al., 2007). ABCA1 functions as a major lipid transporter mediating the cellular efflux of phospholipids and cholesterol, to play an important role in cellular lipid removal (Schmitz and Langmann, 2005). Deficiency of AADAC, a putative triglyceride lipase, contributes to defective lipolysis of cellular triglyceride stores and very-low-density lipoprotein (VLDL) assembly in human hepatocellular carcinoma HuH7.5 cells (Nourbakhsh et al., 2013). Likewise, overexpression of AADAC resulted in significantly reduced intracellular triacylglycerol levels and apolipoprotein B secretion, as well as increased fatty acid oxidation in rat hepatoma cells (Lo et al., 2010). The three genes directly related to lipid metabolism, *MGAT3*, *ABCA1*, and *AADAC* as representative unigenes at the critical juncture of lipid metabolism were targeted by gga-miR-1635 in the ceRNA network as well. Taken together, these results suggest that Z:54674624|54755962 could function as a ceRNA to competitively sponge gga-miR-1635 and novel_miR_232 to regulate the expression of transcriptional factors or genes related to lipid metabolism, such as gga-miR-1635-*AHR2*/*IRF1*/*MGAT3*/*ABCA1*/*AADAC* and/or novel_miR_232-*STAT5A*, during abdominal preadipocyte differentiation and, consequently, regulate adipogenesis in chicken.

Recently, new insights have been gained regarding the protein-coding potential of circRNAs as translational templates to function in various biological processes. Although a covalently closed circular structure without a cap and polyadenylated tail led to the inhibition of cap-dependent translation initiation, the presence of IRES, a cis-acting RNA sequence that mediates the internal entry of 40S ribosomal subunits in eukaryotic cells, might allow the formation of peptides or proteins from circRNAs with effective ORFs (Chen and Sarnow, 1995; Wang and Wang, 2015). As previously reported, circ-PINT, derived from the second exon cyclization of lncRNA-LINC-PINT, is subcellularly located in the cytoplasm and comprises an evolutionarily highly conserved ORF and translates to an 87-amino-acid peptide, PINT87aa, driven by IRES. PINT87aa level was significantly downregulated in tumors and repressed the transcriptional extension of multiple oncogenes by binding to the polymerase-related factor complex gene PAF1, consequently alleviating the occurrence and development of malignant gliomas (Zhang et al., 2018b). Based on circRNA sequencing of myoblasts from human and mouse myotubes, circ-ZNF609, originating from the second exon of the zinc finger protein 609 gene (*ZNF609*), was verified to include a 753 nt ORF, and it also binds to multiple polyribosomes to be translated (Legnini et al., 2017). Another example was that a heart tissue-specific protein translation mechanism derived by ribosome profiling showed that 40 circRNAs might have potential protein translation ability, among which six translational products were qualified via shotgun mass spectrometry (van Heesch et al., 2019). Circ-SHPRH encodes a 17 kDa peptide with a length of 146 amino acids, named SHPRH-146aa, which could protect its parental gene SNF2 histone linker PHD RING helicase (*SHPRH*) from degradation via the ubiquitin proteasome and functions as an inhibitor of glioblastoma (Zhang et al., 2018a). In this study, we predicted the ORF and IRES sequences of circRNAs and suggested that 543 circRNAs (approximately 50%) of all identified circRNAs possessed both IRES and ORF, including 139 circRNAs (approximately 13%) with both IRES and ORFs crossing the BSJ. Almost all were exonic circRNAs, strikingly exceeding the number of both intronic and intergenic circRNAs. This might be explained by the fact that, to a great extent, exonic circRNAs mostly existing in the cytoplasm enable their high protein-coding potential. Regarding the coding potential of intronic and intergenic circRNAs, although they were predominantly enclosed in the nucleus, we speculated that they could shuttle between nuclei and cytoplasm under the facilitation of nuclear export sequences or nuclear localization sequences, such as human miR-29, predominantly localizing in the nucleus (Hwang et al., 2007). Subsequently, the intron circRNAs and intergenic circRNAs undergoing nucleocytoplasmic shuttling could serve as translation templates to harvest peptides and proteins, reflecting the complexity of the translation ability of circRNAs. Although these potentially indicate the possibility of protein-coding potential, their authenticity and mechanisms should still be discovered in further studies.

In conclusion, genome-wide identification of circRNAs in chicken abdominal adipocytes at different differentiation stages was performed using RNA-seq. circRNAs are abundant and differentially expressed and participate in multiple signaling pathways related to lipid metabolism during chicken abdominal preadipocyte differentiation. The feedback expression correlation between specific circRNAs and their parental genes revealed that different types of circRNAs possess diverse regulatory patterns toward parental genes. CircRNAs could function as ceRNAs to efficiently sponge miRNAs to regulate target gene expression and consequently mediate adipogenic differentiation in chickens. Additionally, some circRNAs could also encode proteins that play roles in chicken adipogenesis. Furthermore, two circRNAs, Z:35565770|35568133 and Z:54674624|54755962, might be related to chicken adipogenic differentiation. Of these, Z:35565770|35568133 could negatively regulate the mRNA expression of its parental gene, *ABHD17B*. Z:54674624|54755962 might serve as a miRNA sponge to directly or indirectly regulate chicken abdominal adipocyte differentiation by competitively binding to miR-1635 related to *AHR2*, *IR*F1, *MGAT3*, *ABCA1*, and *AADAC* genes and/or to novel_miR_232 related to *STAT5A*. To the best of our knowledge, our findings are the first to elucidate the identification, characteristics, dynamic expression profile, and potential regulatory roles of circRNAs, not only extending the scope of lipid-relevant circRNAs but also shedding light on a deeper understanding of their functions in avian adipogenesis.

## Data Availability

All RNA-seq data supporting the results of this article have been submitted to National Center for Biotechnology Information (NCBI) Sequence Read Archive (SRA) database under accession number PRJNA732104.
