# Supplementary material for: Dynamic Expression and Regulatory Network of Circular RNA for Abdominal Preadipocytes Differentiation in Chicken (Gallus gallus)
Source: Front Cell Dev Biol. 2021 Nov 12;9:761638. doi: 10.3389/fcell.2021.761638 (PMC8633312; doi:10.3389/fcell.2021.761638)
Supplement: Supplementary file 1 [file DataSheet2.PDF]

Supplementary Material

Supplementary Figures

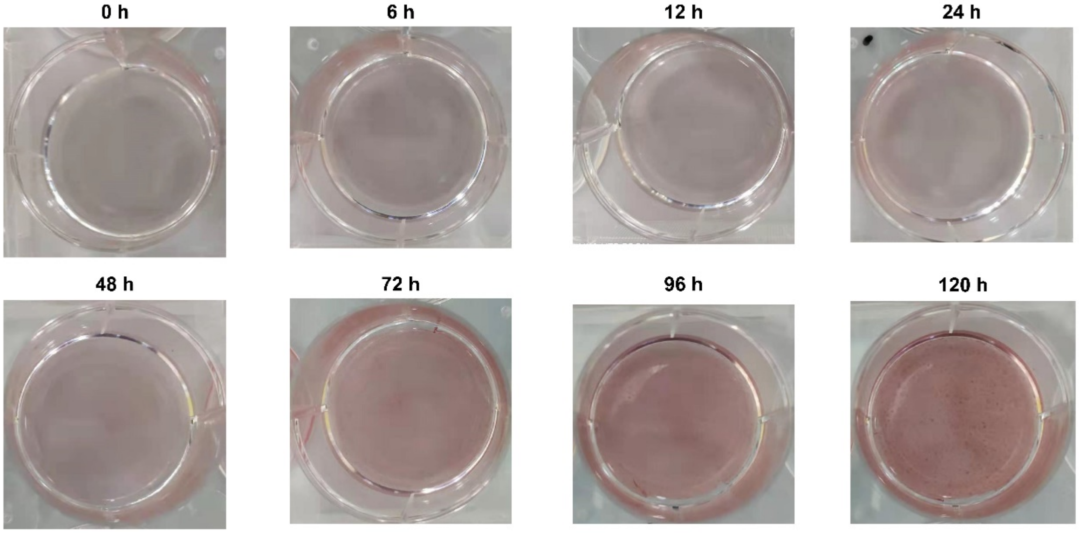

Supplementary Figure 1. Whole cell culture dish views of the Oil Red O staining of chicken abdominal adipocytes at 0 h, 6 h, 12 h, 24 h, 48 h, 72 h, 96 h and 120 h after differentiation.

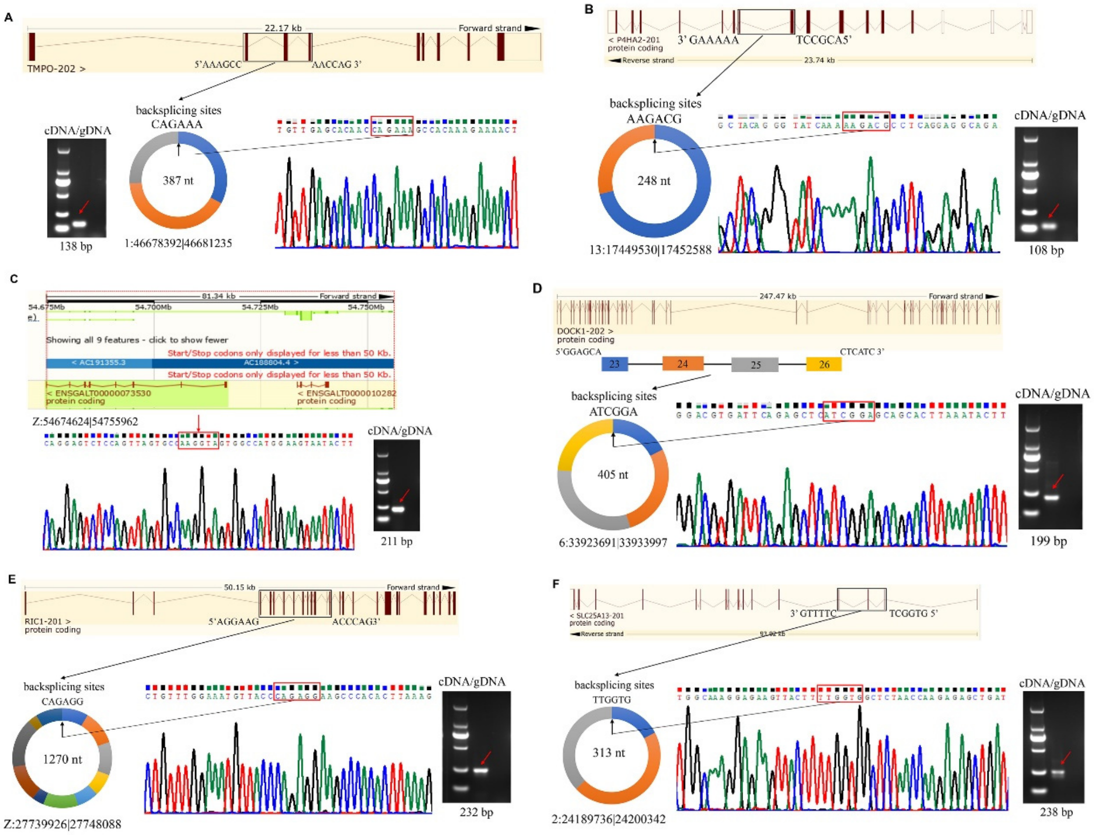

Supplementary Figure 2. Experimental validation of circRNAs. Divergent primers amplify circRNAs using cDNA as templates, but not gDNA. Sanger sequencing confirmed the back-splicing junction sequence of circRNAs.

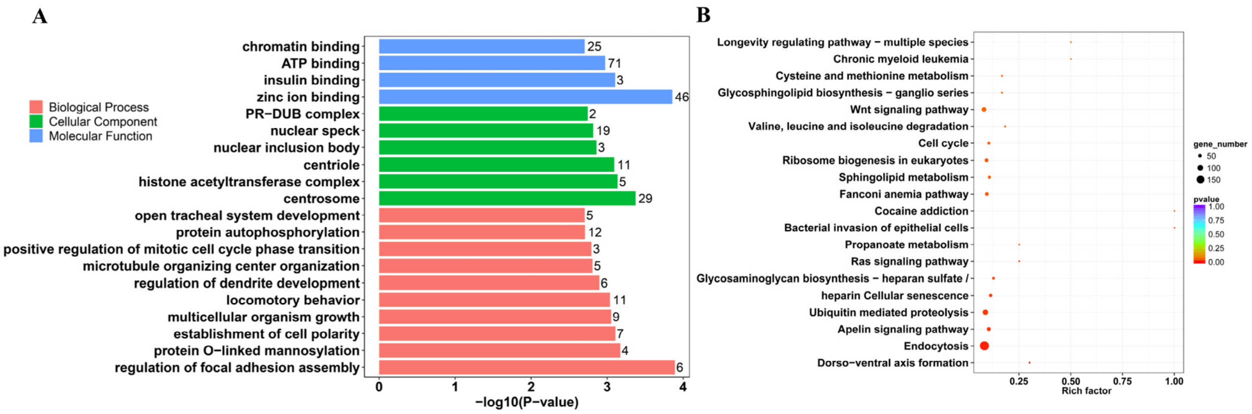

Supplementary Figure 3. GO annotation and KEGG enrichment analysis of parental genes of all identified circRNAs. (**A**) Top 20 significantly enriched GO terms of all identified circRNAs in the biological process, cellular component, and molecular function. (**B**) Top 20 KEGG pathways of all identified circRNAs.

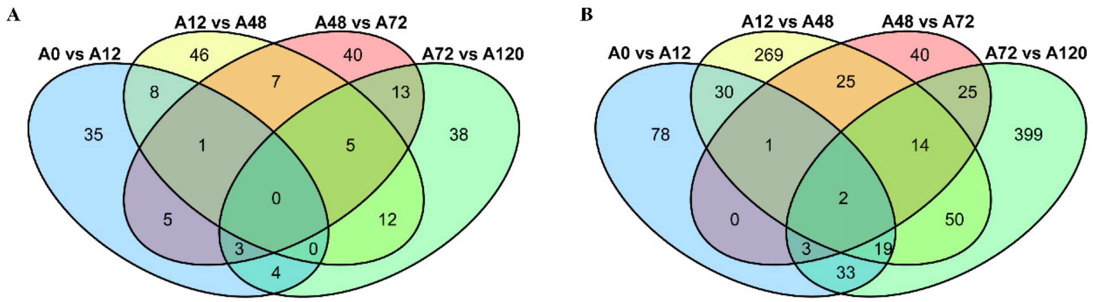

Supplementary Figure 4. Veen analysis of DE-miRNAs **(A)** and DE-mRNAs **(B)** in the four comparisons.

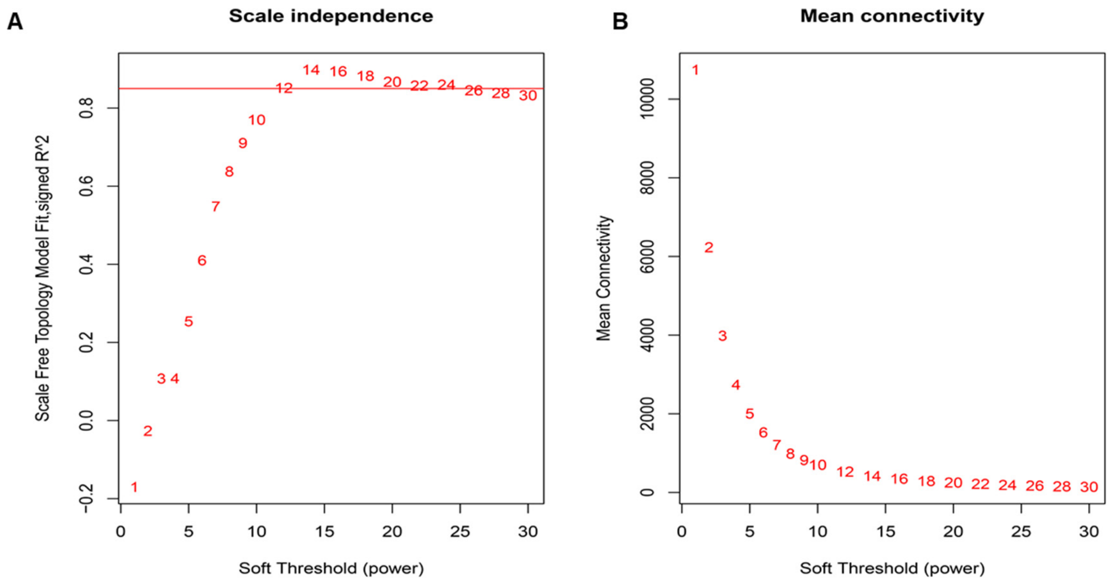

Supplementary Figure 5. Analysis of network topology for soft-thresholding powers from 1 to 30. (**A**) The scale-free fit index (y-axis) is a function of the soft-thresholding power (x-axis). (**B**) The mean connectivity (degree, y-axis) is a function of the soft-thresholding power (x-axis).

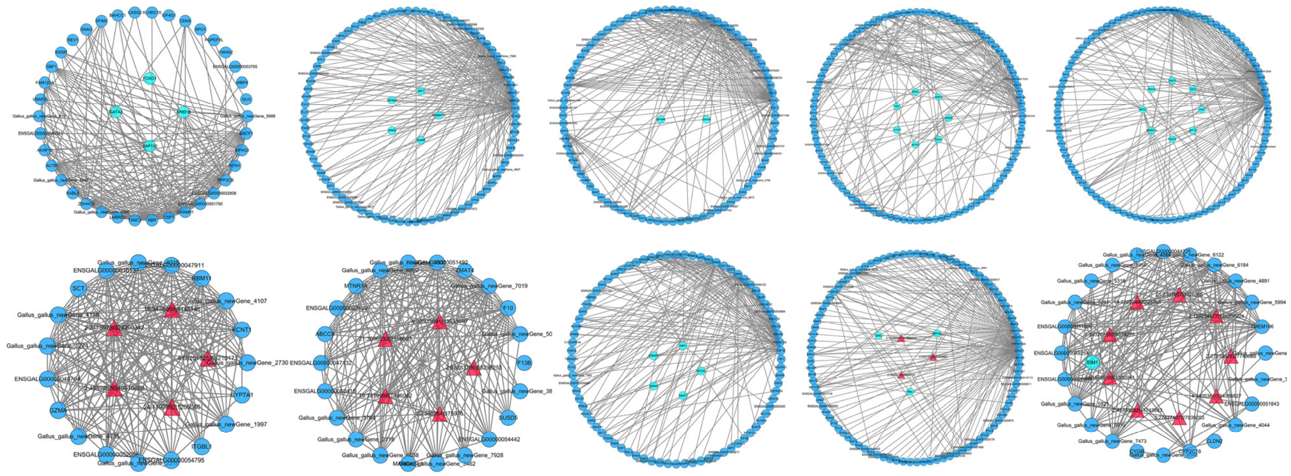

Supplementary Figure 6. Cytoscape visualization of the connected genes with top 200 connectivity in 10 interesting modules. From left to right: “light yellow”, “yellow”, “dark green”, “green”, “turquoise”, “orange”, “dark grey”, “red”, “blue”, “dark turquoise”, respectively. The triangle filled with red represents circRNAs, the circles filled with turquoise and blue represents transcription factor and unigenes, respectively.
